# Biophysical characterization and solution structure of the cannulae-forming protein CanA from the hyperthermophilic archaeon *Pyrodictium abyssi*

**DOI:** 10.1038/s41598-025-13242-6

**Published:** 2025-08-05

**Authors:** Claudia E. Munte, Raphael Kreitner, Reinhard Rachel, Karl O. Stetter, Werner Kremer, Hans Robert Kalbitzer

**Affiliations:** 1 Institute of Biophysics and Physical Biochemistry, Biophysics I and Centre of Magnetic Resonance in Chemistry and Biomedicine (CMRCB), University of Regensburg, Universitätsstr. 31, 93053 Regensburg, Germany; 2 Centre for Electron Microscopy, University of Regensburg, Universitätsstr. 31, 93053 Regensburg, Germany; 3 Lehrstuhl für Mikrobiologie und Archaeen-Zentrum, University of Regensburg, Universitätsstr. 31, 93053 Regensburg, Germany

**Keywords:** Biochemistry, Biophysics, Biotechnology, Microbiology, Structural biology

## Abstract

CanA from *Pyrodictium abyssi*, the main constituent of the extracellular protein network of this archaeon, forms a hollow-fiber network in the presence of divalent ions. The polymerization of CanA induced by divalent ions is characterized by (at least) two processes with rate constants of 0.19 and 0.03 ms^-1^ at 298 K with a critical monomer concentration of 2.48 µM. A non-polymerizing mutant, K_1_-CanA, was created, and the NMR solution structure could be determined by multidimensional NMR spectroscopy. It mainly consists of β-pleated sheets and 2 small α-helices, arranged as β_1_β_2_β_3_β_4_α_1_β_5_β_6_α_2_β_7_β_8_β_9_β_10_β_11_β_12_β_13_. Of the 13 β-strands, 8 form a non-canonical jellyroll class I fold. Several interaction sites for divalent ions could be identified by [^1^H, ^15^N]-SOFAST-HMQC spectroscopy in two main surface areas called BA1 and BA2, located at both ends of the jellyroll. The binding of divalent ions to the monomer induces significant local structural changes in these areas. In general, the affinities for Mg^2+^-ions to the sites in BA1 are smaller than those for Ca^2+^-ions. In contrast, in binding area BA2, Mg^2+^- and Ca^2+^-affinities are similar. The data suggest a conformational selection mechanism induced by ion binding as a first step in the polymerization process of CanA.

**Supplementary Information:**

The online version contains supplementary material available at 10.1038/s41598-025-13242-6.

## Introduction

The hyperthermophilic archaeon *Pyrodictium abyssi* had been isolated from the wall of a black smoker chimney located at a depth of 2005 m below sea level within the Guaymas Basin, Gulf of California^1^. All members of *Pyrodictium* exhibit extremely high optimal growth temperatures between 371 and 378 K^1–3^, and produce unique, complex extracellular matrices that connect the cells. The matrix is formed by hollow fibers called cannulae, which mainly consist of helically arranged glycoproteins and connect the periplasmic space of different cells^4–6^. According to electron microscopy studies, cannulae have an outer diameter of approximately 25 nm, an inner diameter of roughly 20 nm, and a length of up to 150 µm^5–7^.

Three highly homologous *Pyrodictium abyssi* glycoprotein subunits CanA, CanB, and CanC^8^ are found in the cannulae. CanA has a molecular mass of 19.8 kDa and consists of 182 amino acids. CanB and CanC are smaller than CanA and have molecular masses of 15.6 kDa and 16.7 kDa, respectively. An additional N-terminal signaling sequence of 25 amino acids is required for the transport across the membrane to the periplasm of the cells. Sequence homology to other proteins cannot be found in the database. Polymerization of these tubules is coupled to cell division, and the daughter cells stay connected after division.

In *Escherichia coli* expressed CanA spontaneously forms stable tubules in the presence of divalent ions with the same characteristics as native cannulae. Polymerization of CanA can be induced by divalent metal ions such as Mg^2^, Ca^2+^, Cu^2+^, and Zn^2+^. According to the IASPO, reference seawater with 3.5% salinity contains 10.5 mM Ca^2+^, 54.0 mM Mg^2+^, 455.4 mM Na^+^, and 10.2 mM K^+^^9^. Optimal cannulae formation can already be obtained at somewhat lower concentrations of divalent ions, e.g., by adding 10 mM CaCl_2_ and 10 mM MgCl_2_^8^.

**Fig. 1:**
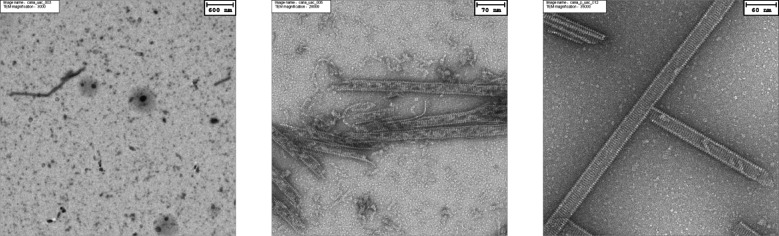
TEM of recombinant CanA. (Left) Overview of “monomeric” CanA under standard NMR conditions (2 mM CanA in 50 mM Tris/HCl, pH 6.5, 80 mM NaCl, 0.4 mM NaN_3_) incubated for approximately 12 h, (middle) detection of sparse cannulae in this sample, (right) ^15^N-enriched, biosynthetically produced CanA after polymerization in the presence of 10 mM CaCl_2_ and 10 mM MgCl_2_ for 12 h. Temperature 303 K.

Cannulae formed by biosynthetically produced, pure CanA monomers are stable up to 401 K, that is their temperature stability is similar to that observed for cannulae prepared from natural sources. However, the latter contain also other variants of cannulae forming proteins than CanA and are glycosylated^5–7^.

Limited trypsin digestion of CanA led to the identification of K_1_-CanA where the 10 N-terminal residues are removed^10^. This construct, when biosynthetically produced, gives perfect NMR spectra at 323 K, and is stable for extended periods (weeks and even many months). An almost complete homo- and heteronuclear assignment of K_1_-CanA has been reported by Kreitner et al.^10^. The secondary structure could be derived from an analysis of the C′, C^α^, C^β^, N, H^N^, and H^α^ chemical shifts. It predicts 6% helices, 44% β-pleated sheets, and 50% coils for K_1_-CanA. Most recently, a cryo-EM structure of CanA cannulae has been deposited in the PDB database (accession # 7UII).

The study and modification of carbon nanotubes (CNTs) is a central research topic in nanophysics. Hollow fibers with inner diameters between 50 and 150 nm can be produced nowadays and show promising physical features (see e.g.^11,12^). CanA nanotubes (cannulae) have similar inner diameters and could become the corresponding material in nanobiophysics because of their stability and heat resistance. Compared to carbon nanofibers, the introduction of new features in the CanA nanotubes by biotechnological means appears to be straightforward and relatively easy when the three-dimensional structure is known in atomic detail.

In this paper, the specific biophysical properties of CanA and its truncated form K_1_-CanA, together with the solution structure of monomeric K_1_-CanA determined by NMR, will be reported and used for the interpretation of the formation of cannulae induced by divalent ions. It is also a prerequisite for understanding the three-dimensional structure of cannulae themselves.

## Results

### Structure of K_1_-CanA and CanA

TEM micrographs were produced from CanA expressed in *E. coli *and incubated for 12 h in the absence or presence of divalent ions. For experimental details, see Materials and Methods. A selection of the micrographs is presented in Fig. 1. The left panel of Fig. 1 shows the TEM micrograph of recombinant CanA after incubation at 303 K for approximately 12 h in the absence of divalent ions, where mainly monomers and small aggregates can be observed even in the absence of divalent ions. The middle panel shows rare cannula-like polymers that also can be observed under these “non-polymerization” conditions at higher magnification. After induction of polymerization by addition of 10 mM CaCl_2_ and 10 mM MgCl_2_ for 12 h at 303 K (Fig. 1, right), the cannulae show a very regular, well-defined pattern, their outer diameter lies between 25 and 35 nm and is thus somewhat larger than that of natural cannulae (25 nm). The periodicity is 4.4 nm^-1^, the helical slope 7° to 8°. The subunits are arranged in a two-stranded helix with a periodicity of (4.4 nm)^-1^.

**Fig. 2:**
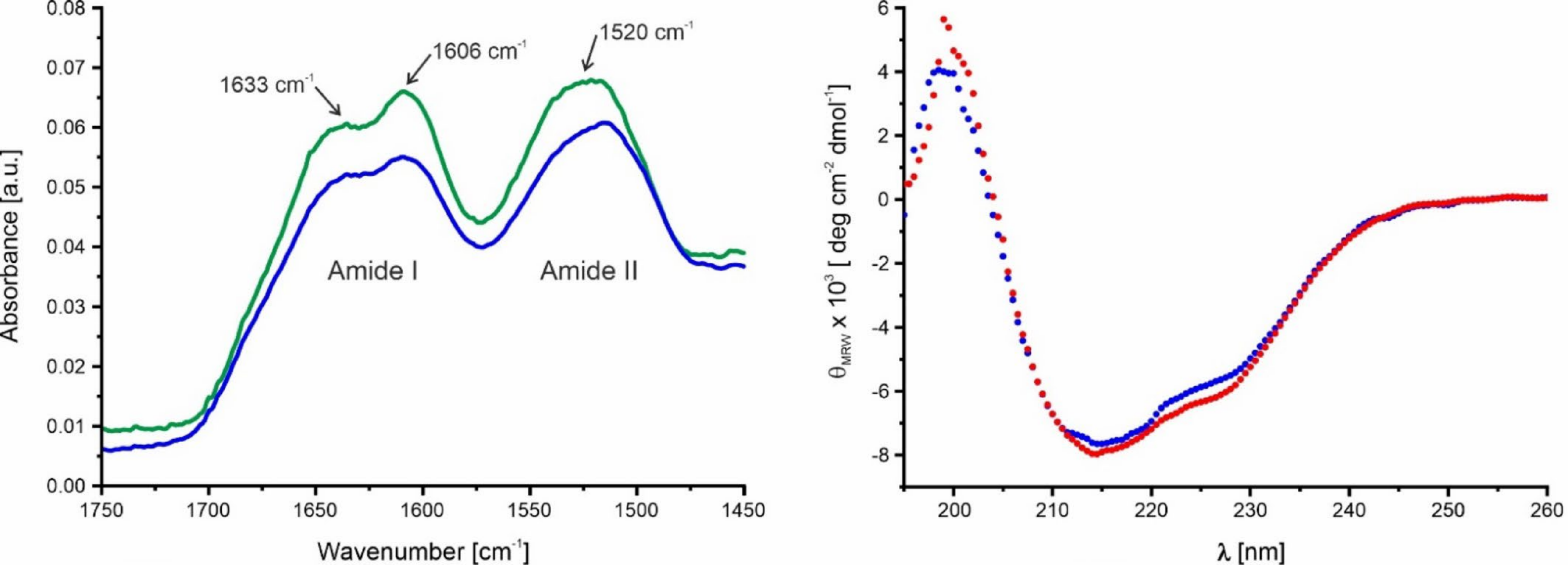
FT-IR and CD spectra of CanA. (Left) FT-IR spectra from the monomeric CanA (blue) and CanA polymerized by addition of 10 mM CaCl_2_ and 10 mM MgCl_2_ for 12 h (green). The sample contained approximately 1 mg of protein dissolved in 5 µL of H_2_O. (Right) CD spectra of 1 mg/mL CanA (blue) or K_1_-CanA (red) in H_2_O, pH 7.0, at 298 K.

In principle, the polymerization of CanA could induce large scale structural changes of the CanA monomer. FT-IR shows that after polymerization, the general three-dimensional structure of CanA monomers is essentially preserved (Fig. 2, left). The N-terminal truncation of CanA in K_1_-CanA could also cause larger conformational changes of the monomeric units. However, CanA and K_1_-CanA have almost identical CD spectra, indicating that their secondary structures are almost identical (Fig. 2, right). The secondary structure analysis of the data using the program CDSSTR^13^ gives very similar propensities for CanA and K_1_-CanA (α-helix 4%, and 5%; β-sheet 42% and 41%; loops 23% and 22%; disordered 30% and 31%). They are close to the values obtained by the TALOS-N^14^ secondary structure prediction from the H^N^, H^α^, N, C’, C^α^, and C^β^ chemical shifts of Can A and K_1_-CanA^10^ (α-helix 6%; β-sheet 41% and 44%; coils 53% and 50%).

**Fig. 3:**
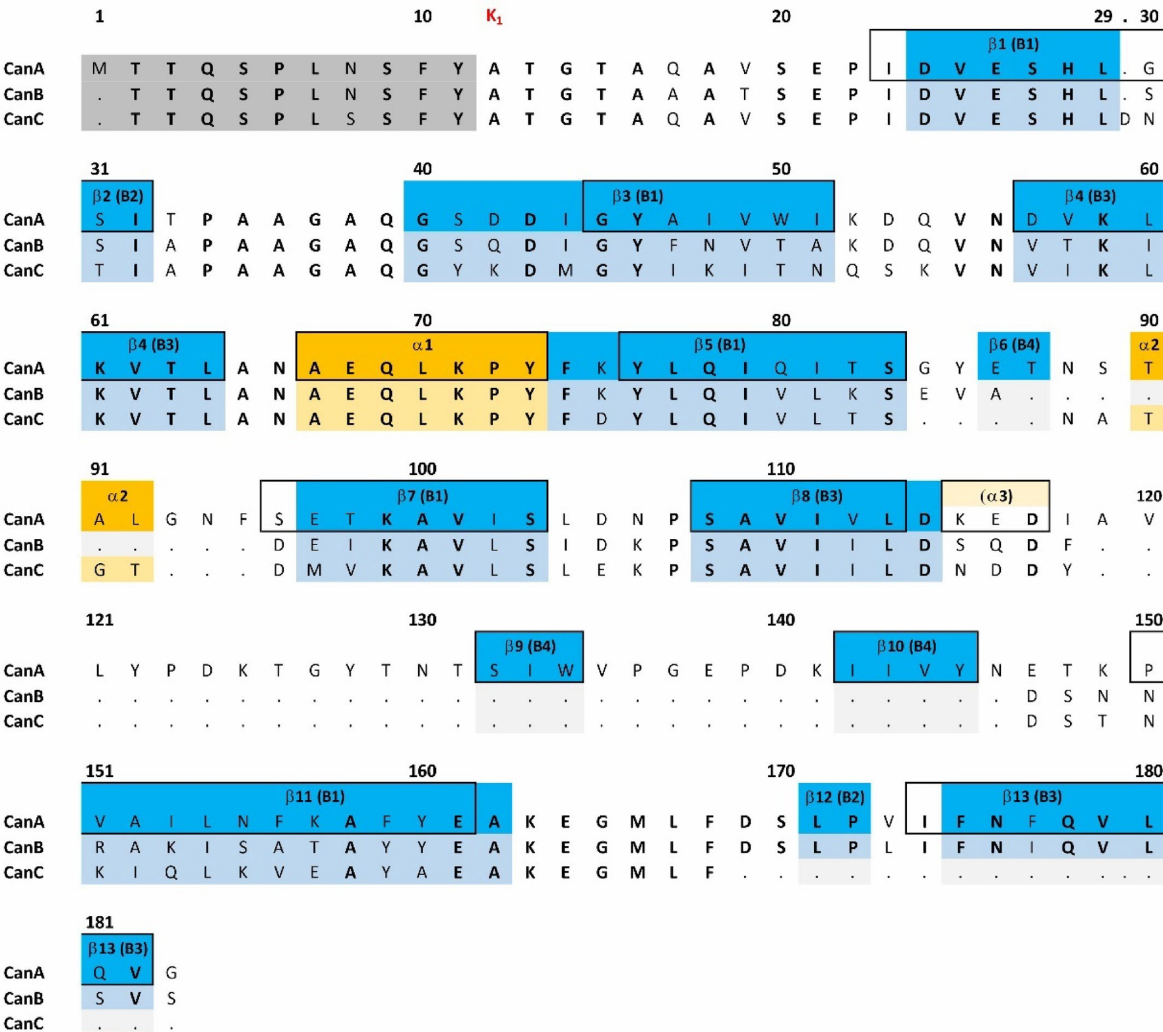
Amino acid sequence and secondary structure of CanA, CanB, and CanC. Amino acids removed in K_1_-CanA (grey), β-strands recognized in the 3D-NMR structure (blue), α-helices recognized in the 3D-NMR structure (orange), and β-strands or α-helices recognized by chemical shift analysis (boxed)^10^. Predicted helix α3 is only observed in one of the 10 lowest energy structures. CanA, CanB, and CanC sequences are taken from Mai^8^. Note that the N-terminal methionine M1 of CanA is introduced by the recombinant expression in *E. coli* and is not contained in the natural protein (and in the sequence presented by Kreitner et al.^10^).

Nearly complete backbone and side chain assignments, including a large number of stereospecific assignments, were obtained for K_1_-CanA, the N-terminally truncated form of CanA as well as a secondary structure prediction from the H^N^, H^α^, N, C’, C^α^, and C^β^ chemical shifts^10^. 10 β-strands and 2 α-helices had been predicted in K_1_-CanA by the program TALOS-N from these data. The amino acid sequence of CanA and its alignment with CanB and CanC are shown in Fig. 3. The secondary structure elements predicted from backbone chemical shifts are depicted in the CanA sequence (boxed residues).

The structure of K_1_-CanA was calculated using 16.5 restraints per residue by restrained molecular dynamics and simulated annealing using the standard protocols from CNS. Supplementary Table S1 summarizes the restraints used for the structure calculations. All peptide bonds were assumed to occur in the *trans*-configuration as analyzed by Kreitner et al.^10^. From the 1000 structures calculated, the 10 structures with the lowest total energy were selected for further analysis. Supplementary Table S2 shows the corresponding structural statistics.

The calculated 10 lowest energy structures of K_1_-CanA show a high structural similarity, reflecting both the excellent quality of the spectra and the relatively high number of structural restraints (Supplementary Tables S1 and S2). In the Ramachandran plot of the backbone dihedral angles, the majority (97%) of (ϕ, ψ)-angles are found in the favored region, 2% in the allowed region, and 1% in the disallowed region.

**Fig. 4:**
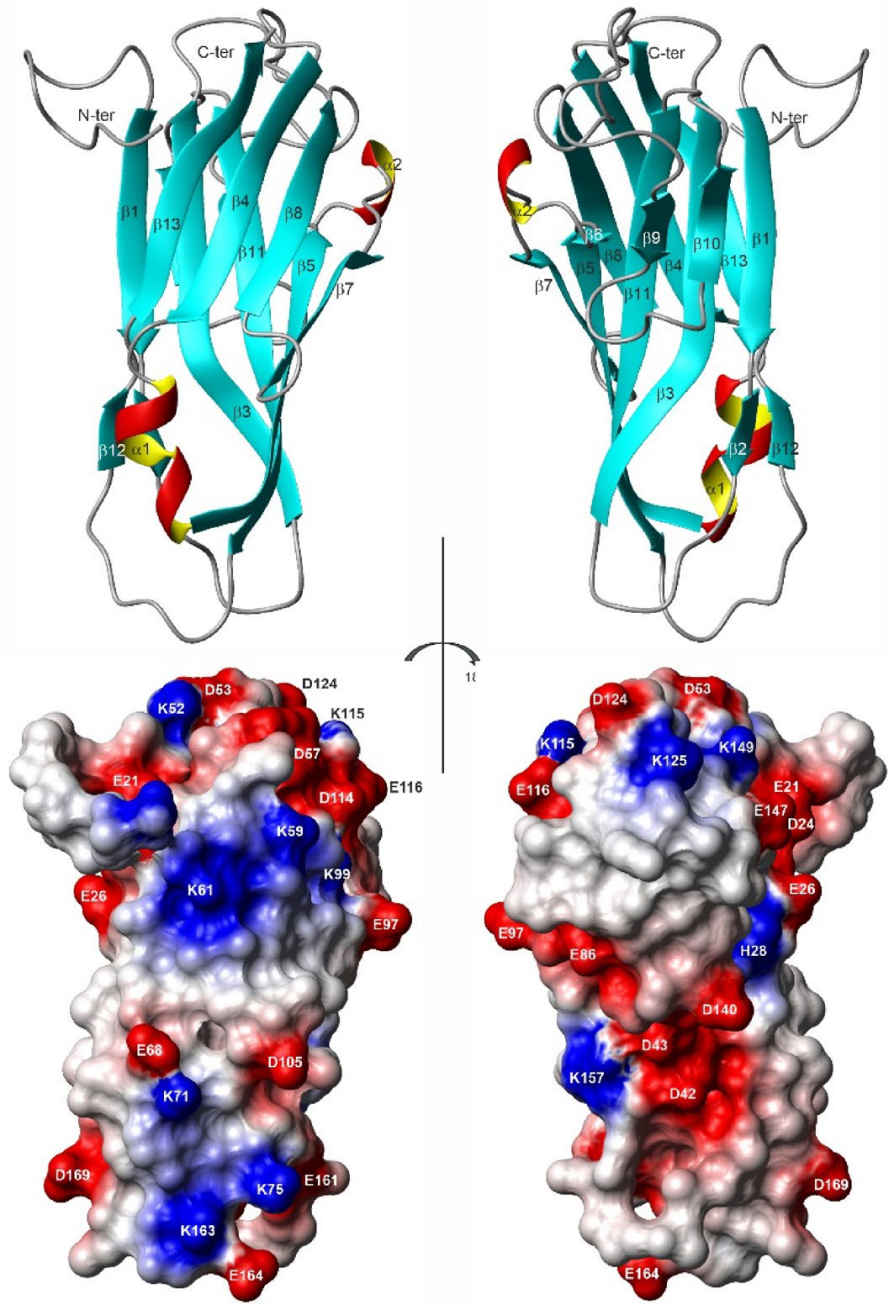
NMR structure of K_1_-CanA from *Pyrodictium abyssi*. (Top) Lowest energy structure in ribbon representation. (Bottom) Surface of the protein depicting the electric potentials (negative, red; positive blue). The residues with charged side chains are labeled. Note that the N-terminal methionine (M11) originating from the protein expression is not shown and was not introduced in the structural calculation. Based on the cryo-EM structure 7UII, the NMR structures are viewed from the interior (left) and the exterior (right) of the cannulae. Structures were represented by MolMol^15^.

The location of the secondary elements is indicated in Fig. 3. The three-dimensional arrangement of secondary structure elements in K_1_-CanA is depicted in Fig. 4. A β_1_β_2_β_3_β_4_α_1_β_5_β_6_α_2_β_7_β_8_β_9_β_10_β_11_β_12_β_13_ secondary structure pattern can be derived from the three-dimensional structure (Fig. 4). Two small helices and four antiparallel β-sheets are present in the structure. The helices encompass residues Ala67-Tyr73 (α_1_), and Thr90-Leu92 (α_2_). In one of the 10 deposited structures, a third short helix encompassing residues Lys115-Asp117 (α_3_) is present. The five-stranded β-sheet B1 comprises residues Asp24-Leu29 (β_1_), Gly40-Ile51 (β_3_), Phe74-Ser83 (β_5_), Glu97-Ser103 (β_7_), and Val151-Ala162 (β_11_). The three-stranded β-sheet B3 is formed by residues Asp57-Leu64 (β_4_), Ser108-Asp114 (β_8_), and Phe175-Val182 (β_13_). Residues Ser31-Ile32 (β_2_), and Leu171-Pro172 (β_12_) form the short two-stranded β-sheet B2; residues Glu86-Thr87 (β_6_), Ser132-Trp134 (β_9_), and Ile142-Tyr145 (β_10_) the short three-stranded β-sheet B4. This is in good accordance but not identical with the TALOS-N (Shen et al. 2013) predictions (see Fig. 3). However, as usual, the secondary structure predictions do not completely coincide with those found in calculated 3D-structures (Fig. 3). Nevertheless, these differences can give some additional information, since they are usually interpreted as rare conformational states with somewhat higher free energies.

The three-dimensional structure of K_1_-CanA is depicted in Fig. 4. The electric field on the surface of the protein and the corresponding charged residues are also depicted. The charged residues and the corresponding electric field distribution are not equally distributed but are locally clustered. In Fig. 4, the structures are oriented in such a way that the surface oriented to the interior and the exterior of the cannulae is visible (see below). It shows that inside the cannulae positively charged residues and outside negatively charged residues are predominant. As a consequence, electric field components directed from the inside to the outside of the cannulae would be expected when they are not compensated by residues from neighboring protomers in the multimeric structure.

**Fig. 5:**
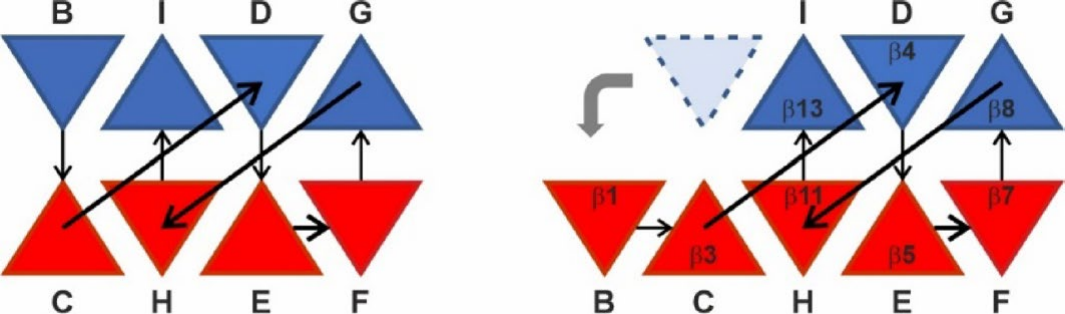
Triangle diagram of the jellyroll topology. The strands comprising the two β-sheets are shown in different colors. The relative orientations of the triangles indicate that the sheets are antiparallel. (Left) The topology observed in a class I jellyroll which is characterized by the first strand (B) being an edge strand (different from class II in which the first strand is internal). (Right) The modified topology observed in K_1_-CanA in which strand B has migrated from the blue sheet to the red sheet.

Both the five-stranded β-sheet B1 and the three-stranded β-sheet B3 are arranged in a non-canonical jellyroll class I fold, identified by visual inspection by an expert in the field (see Acknowledgments). In a canonical jellyroll class I, the sheet is composed of strands BIDG and CHEF, folded such that strand B packs opposite strand C, I opposite H, etc., as schematically shown in Fig. 5^16^. In the CanA strand B jumped to the left of strand C, leading to a composition of strands IDG (β_13_, β_4_, β_8_) and BCHEF (β_1_, β_3_, β_11_, β_5_, β_7_) (Fig. 5). This variation does not appear to be a mere structural detail but rather functionally important for the protein, probably for the formation of the cannulae. The β-sheets B2 (β_2_, β_12_) and B4 (β_6_, β_9_, β_10_) do not belong to the jellyroll and are probably also important in the cannulae formation.

### Oligomer and polymer formation of CanA and K_1_-CanA

Under the experimental conditions typically used for the structure determination, freshly prepared full-length CanA gives well-resolved two-dimensional NMR spectra. However, after longer incubation at 313 K, the overall intensity of the [^1^H, ^15^N]-cross peaks is reduced with time. After correction of the overall signal reduction by renormalization, some resonances get weaker or disappear completely, and a few very weak, new peaks appear with time. This is probably due to two different mechanisms, to a weak specific protein–protein interaction (oligomerization) and to some polymerization of the initially monomeric CanA as it has been observed also by electron microscopy (Fig. 1). In line with this hypothesis, removal of higher molecular mass complexes by gel filtration of the sample leads to the recovery of the initial spectrum with an abolishment of the time-dependent sequence specific spectral changes.

Since the quality of the NMR spectra deteriorates with time, full-length CanA itself is not well-suited for NMR structure determination. Multidimensional NMR spectroscopy requires monomeric samples stable for several days. We have already reported the NMR assignments of the truncated CanA variant K_1_-CanA identified by limited proteolysis^10^. Here, the first 11 amino acids were removed from the recombinant protein. With this construct, the 3D structure in solution could be performed successfully (see above). Note that recombinant CanA as well as recombinant K_1_-CanA, contain an additional N-terminal methionine required for protein expression.

**Fig. 6:**
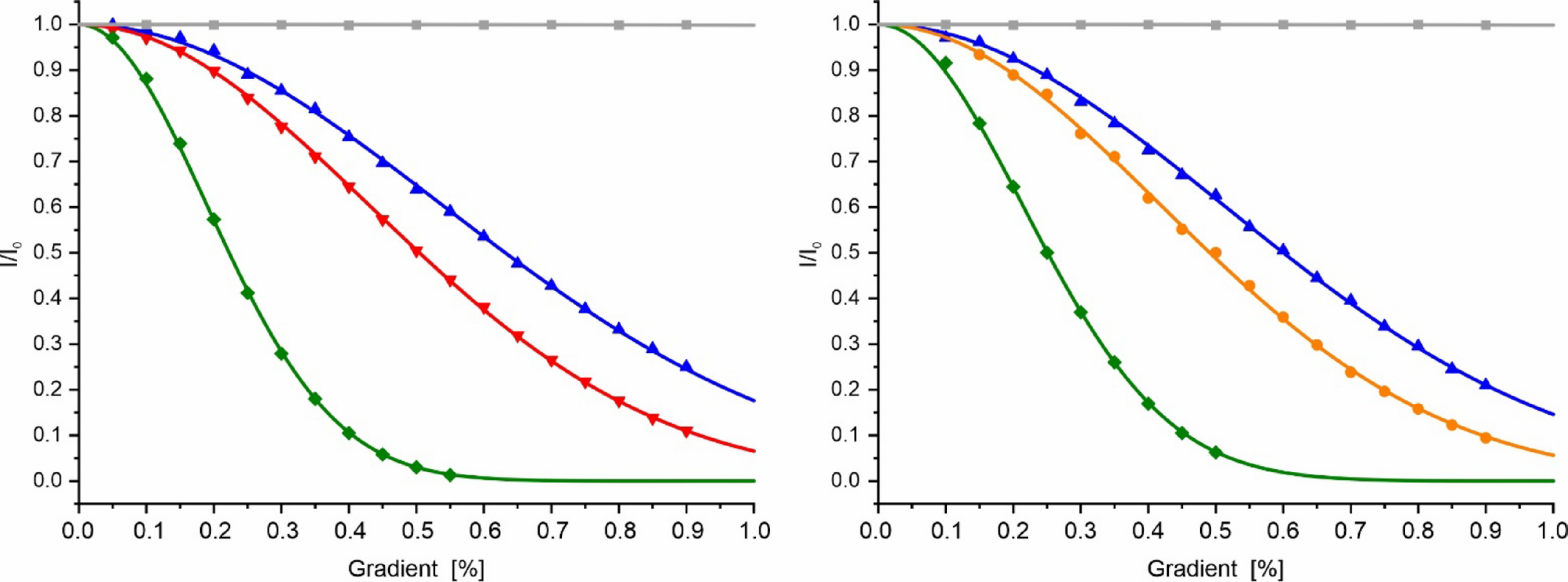
Effect of pulsed magnetic field gradients on the NMR spectra of CanA. The samples contained 0.5 mM CanA in 10 mM phosphate buffer, 0.1 mM DSS, 1 µM EDTA. The relative signal intensities *I*/*I*_0_ are plotted as a function of the gradient strength. Temperature 318 K. For more experimental details, see Materials and Methods. DSS (green) and 20% polyacrylamide (grey). (Left) Sample of CanA incubated for 6 h at pH 6.6 (red) or pH 9.0 (blue); (right) sample of CanA at pH 9.0 before (blue) and after (orange) gel filtration.

Weakly interacting proteins can be characterized by NMR diffusion measurements since the interaction influences the apparent diffusion constants measured. Figure 6 shows the signal intensity dependence on the strength of the z-gradient of the pulsed magnetic field for CanA. The samples contained DSS as a reference. Before and after every experiment, the effects of the pulsed field gradients on polymerized acrylamide were measured to exclude unspecific gradient effects. Under optimal experimental conditions, no gradient effects should be visible, as is the case here (Fig. 6).

When CanA was incubated at pH 6.6 or pH 9.0 for 6 h at 318 K, apparent molecular masses of 28.20 kg/mol and 64.63 kg/mol, respectively, were obtained. The molecular mass calculated from the amino acid sequence is 19.85 kg/mol. These too-high apparent molecular masses indicate that the diffusion of the CanA monomers is slowed down by interactions with oligomers and/or polymers, as they are also observed by TEM (Fig. 1). This assumption is supported by our NMR diffusion experiments that were performed immediately after gel filtration of the samples. Now, at pH 9, a molecular mass of 22.31 kg/mol is obtained, close to the ideal value. The corresponding hydrodynamic radius is 1.68 nm. Dynamic light scattering gives a value of 1.74 nm (data not shown). Since the increase of the apparent molecular mass is slowly dependent on the time of incubation, most probably, not a simple monomer–monomer interaction is responsible for the increase of the apparent diffusion constants but the interaction of monomers with multimers in different concentrations that are formed with time. This assumption is in line with the TEM results (Fig. 1) which shows different aggregates including cannulae-like structures. As an independent factor, the creation of polymers in the absence of divalent ions is enhanced by increased pH of the solution. Usually in proteins, a pH dependence is created by protonation/deprotonation of histidine residues. CanA only contains one histidine residue (H28) which could be responsible for such a pH-switch. Generally, it can be expected that the exact composition of the CanA sample at a given time is dependent on not completely controllable factors like the existence of seeds. Such an effect has also been observed in the dynamic light scattering experiments, where the average hydrodynamic radius assignable to monomers was well preserved in all experiments, but the size distribution of some additional high molecular mass aggregates varied from experiment to experiment.

**Fig. 7:**
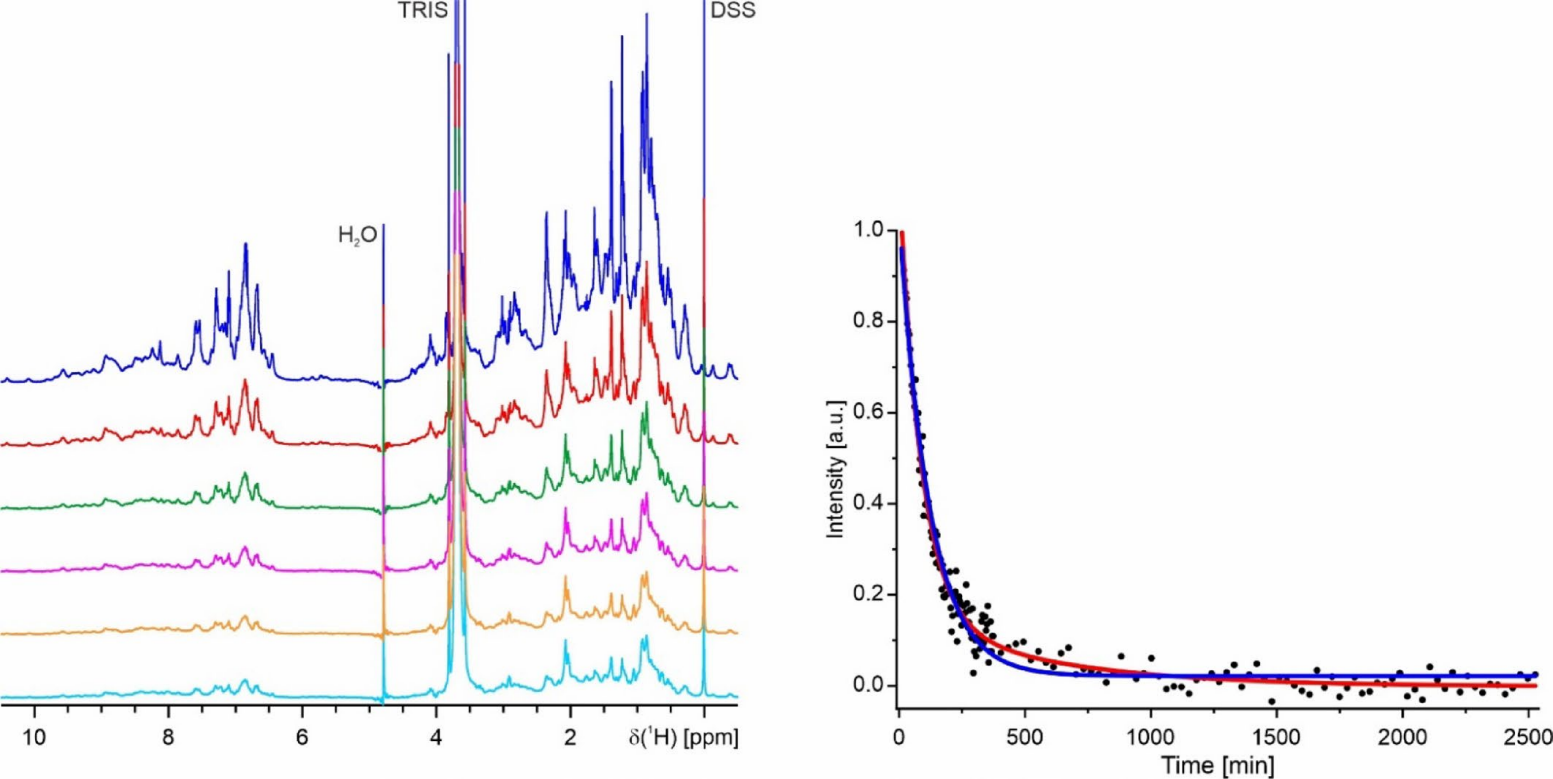
Time course of polymerization of CanA. (Left) ^1^H-spectra of CanA after starting the polymerization at 298 K by the addition of MgCl_2_ and CaCl_2_ up to end concentrations of 20 mM. The sample contained 0.97 mM CanA in 50 mM Tris–HCl, 50 mM NaCl, pH 7.5, 10% D_2_O, 0.4 mM DSS. The top spectrum was recorded directly after the addition of the divalent ions (dark blue), after 1 h 12 min (red), after 2 h 24 min (green), after 3 h 36 min (magenta), after 4 h 48 min (orange), and after 5 h 57 min (light blue). (Right) Fit of the signal intensity in the ppm range (-0.6, 0.1) due to polymerization by a single (blue) or a double (red) exponential decay. The obtained rate constants for a fit with a single exponential *k*_pol_, and a double exponential *k*_pol,1_ and *k*_pol,2_ are 0.14 ms^-1^, 0.19 ms^-1^, and 0.03 ms^-1^, respectively.

Although the CD-spectra (Fig. 2, right) and the NMR spectra (see below), and hence the folding of K_1_-CanA and full-length CanA are very similar, their behavior under polymerization conditions is completely different. Figure 7 (left) shows the 1D spectra of freshly dissolved CanA after fast addition of appropriate amounts of a solution of 1 M MgCl_2_ and CaCl_2_ up to end concentrations of 20 mM each. The initial 1D spectrum is typical for a monomeric, well-folded protein of the size of CanA. The spectra were recorded in total for approximately 42 h, the first 100 spectra were recorded with a repetition time of 3.5 min. For the remaining 72 spectra the number of scans has been increased by a factor of 2, and spectra were recorded every 23 min, leading to a total repetition time of 29.5 min per spectrum. The intensities could be normalized by the signal of free DSS. The initial spectrum shows several high-field shifted resonances below 0.1 ppm that correspond to methyl groups of amino acids in the core of the protein in contact with aromatic rings. With time, their intensities (integrals) go down together with the overall intensities and are barely observable after 6 h (Fig. 7, left). Such a behavior would be expected when the initially monomeric protein forms large complexes with long rotational correlation times. In the simplest case, only the spectrum of the monomer can be observed, whereas the resonance lines of the large complexes are too broad to be detectable. The intensities of resonance lines from the rigid parts of the structure are proportional to the concentration of free CanA monomers. When these intensities are plotted as a function of time (Fig. 7, right), it is evident that the data can be better represented by a double exponential than by a single exponential (reduced χ^2^ of 0.00089 and 0.0012, respectively). This indicates the occurrence of at least two different processes during the polymerization. The corresponding rate constants for these two processes are 0.19 ms^-1^ and 0.03 ms^-1^. The asymptotic value 2.48 μM corresponds to the critical CanA concentration for polymerization. During the induced polymerization of isolated monomers to form a complex-structured polymer, several steps with different time constants are expected. A necessary fast step would be the formation of dimers or small oligomers, and a significantly slower step should be organization into cannula-like structures.

**Fig. 8:**
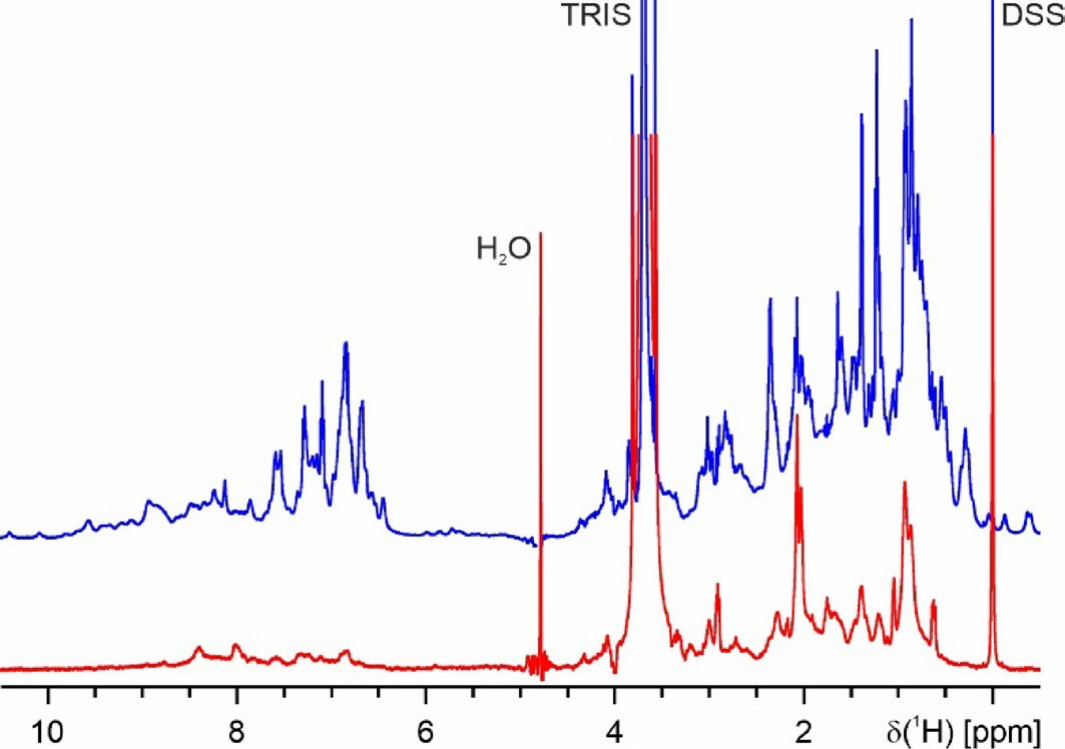
^1^H-NMR spectra of polymerized CanA. (Top) Spectrum recorded directly after addition of the divalent ions, (bottom) spectrum recorded after 42 h. Note that the intensity of the bottom spectrum was scaled up by a factor of 4. For experimental conditions, see Fig. 7.

The ^1^H-NMR spectrum of monomeric full-length CanA contains several sharp resonance lines that cannot be observed in K_1_-CanA with line widths in the range of 5 and 8 Hz. Their chemical shift values and their coupling patterns correspond closely to those expected for amino acids 1 to 11. Directly after the addition of MgCl_2_ and CaCl_2_ (end concentrations of 20 mM) they are somewhat broadened and shifted but remain strongly visible. The other lines in the monomer have linewidths around 20 Hz. Even after 42 h, some weak NMR signals remain visible with linewidths of 20 to 30 Hz (Fig. 8). These resonance lines of the N-terminus are observable at similar positions, thus assigning part of these lines to the 11 N-terminal amino acids of CanA. They may correspond to regions of the protein with high internal mobility (low motional correlation times) that may still be detected in the large multimeric complex with its high rotational correlation time. However, the majority of all resonance lines in monomeric K_1_-CanA and CanA have mean linewidths between 20 and 50 Hz, as expected for a monomeric protein with a molecular mass of 19.8 kDa at 298 K. Methyl groups of the methionines show up as strong singlets around 2 ppm. In the spectra remaining after polymerization, two singlet resonances are still visible at the expected chemical shifts, namely at 2.03 ppm and 2.07 ppm. Natural CanA contains only one methionine (M166). However, our protein expressed in *E. coli* contains one additional N-terminal methionine from the start codon. The intensities of these sharp methyl resonances of the two methionines, as well as some other non-assigned resonances, are still about 30% of their original intensityies in contrast, those of the folded parts have less than 3% of their original intensities after polymerization.

**Fig. 9:**
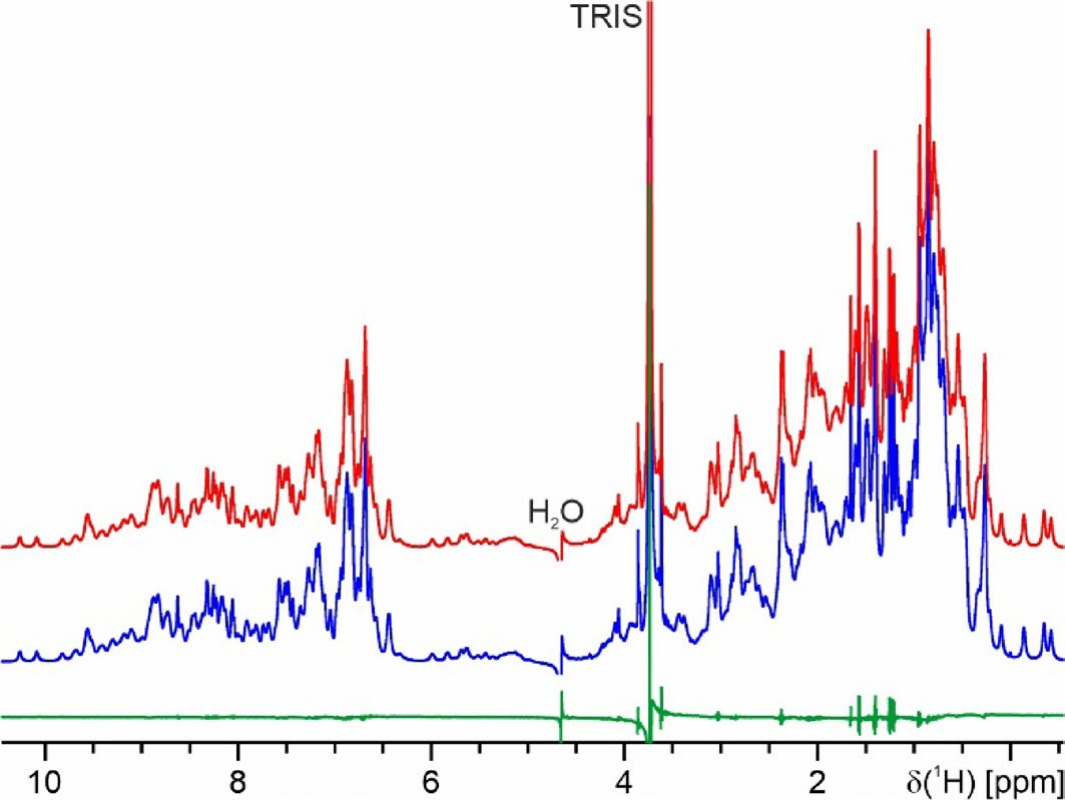
Time dependence of the ^1^H signal intensities of K_1_-CanA. ^1^H-spectra of K_1_-CanA at 310 K after adding 8 mM MgCl_2_ and CaCl_2_. The sample contained 0.1 mM of K_1_-CanA in 50 mM Tris–HCl, 50 mM NaCl, pH 7.5, 10% D_2_O, 0.4 mM DSS. (Blue) Spectrum recorded directly after addition of the divalent ions; (red) spectrum after 20 h.; (green) difference of the two spectra.

The time dependence of the ^1^H signal intensities of K_1_-CanA is completely different. The NMR spectrum is virtually unchanged after 20 h under polymerization conditions, meaning that during this time no polymerization is occurring (Fig. 9). This is also in line with EM data of K_1_-CanA, where no polymers are observed.

### Conformational changes induced by N-terminal truncation

**Fig. 10:**
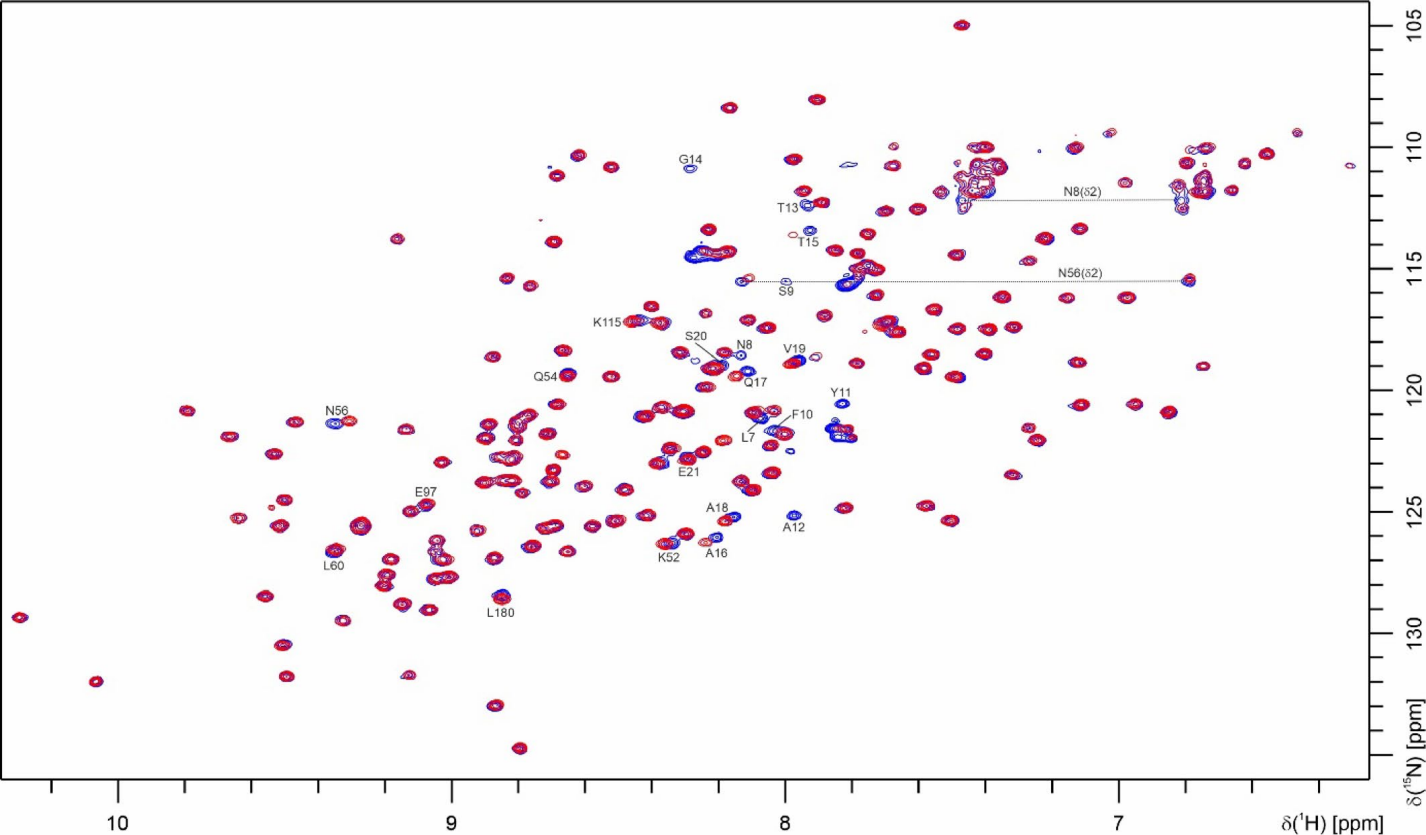
Superposition of [^1^H, ^15^N]-SOFAST-HMQC spectra of ^15^N-enriched CanA and K_1_-CanA. Spectra from CanA (blue), and K_1_-CanA (red). Temperature 323 K, 800 MHz proton frequency. 0.14 mM protein, 50 mM TRIS/HCl, pH = 6.6, 80 mM NaCl, 10% D_2_O, 0.4 mM DSS.

The NMR spectra of full-length and truncated CanA monomers are very similar and indicate a very high degree of structural similarity. Figure 10 shows a superposition of the amide fingerprint region of ^15^N-enriched CanA and K_1_-CanA. The [^1^H, ^15^N]-SOFAST-HMQC spectra presenting the amide cross peaks of the two proteins are almost identical. Most but not all resonances have identical shifts in the two spectra. Compared to K_1_-CanA, additional peaks are observed in the CanA spectrum at 323 K (Fig. 10) that partly originate from the 10 additional N-terminal amino acids in CanA and could be assigned by 2D methods. In CanA L7, N8, S9, F10, Y11, A12, T13, and G14 are visible and could identified. T2, T3, Q4, and S5 of the N-terminus do not give strong enough HMQC signals (Table 1) at this temperature. At position 6 is a proline residue that does not have an amide group and therefore, is also not visible in the HMQC spectrum. Analogously, in the N-terminus of K_1_-CanA, the HMQC signals of the first amino acids (A12, T13, G14) are too weak to be observable.

**Table 1:** Chemical shifts of the mobile N-terminus of CanA at 313 K and 323 K^a^.

Residue	T2	T3	Q4	S5	L7	N8	S9	F10	Y11	A12
313 K										
δ(H^N^) [ppm]	8.134	8.150	8.455	8.339 (8.023)	8.139 (8.177)	8.187	8.054	8.091 (8.235)	7.880 (7.849)	8.033 (7.992)
δ(N^H^) [ppm]	119.3	116.2	123.3	118.9 (116.8)	121.2 (121.8)	118.6	115.5	121.7 (121.9)	120.6 (121.1)	125.2 (126.0)
323 K										
δ(H^N^) [ppm]	-^b^	-^b^	-^b^	-^b^	8.073	8.132	7.998	8.030	7.828	7.973
δ(N^H^) [ppm]	-^b^	-^b^	-^b^	-^b^	121.2	118.6	115.5	121.7	120.5	125.1

^a^Sample contained 0.14 mM CanA in 50 mM TRIS/HCl, pH = 6.6, 80 mM NaCl, 10% D_2_O, 0.4 mM DSS. Values in brackets, second conformer.

^b^Resonances not observed in the [^1^H, ^15^N]-SOFAST-HMQC spectrum at 323 K.

**Fig. 11:**
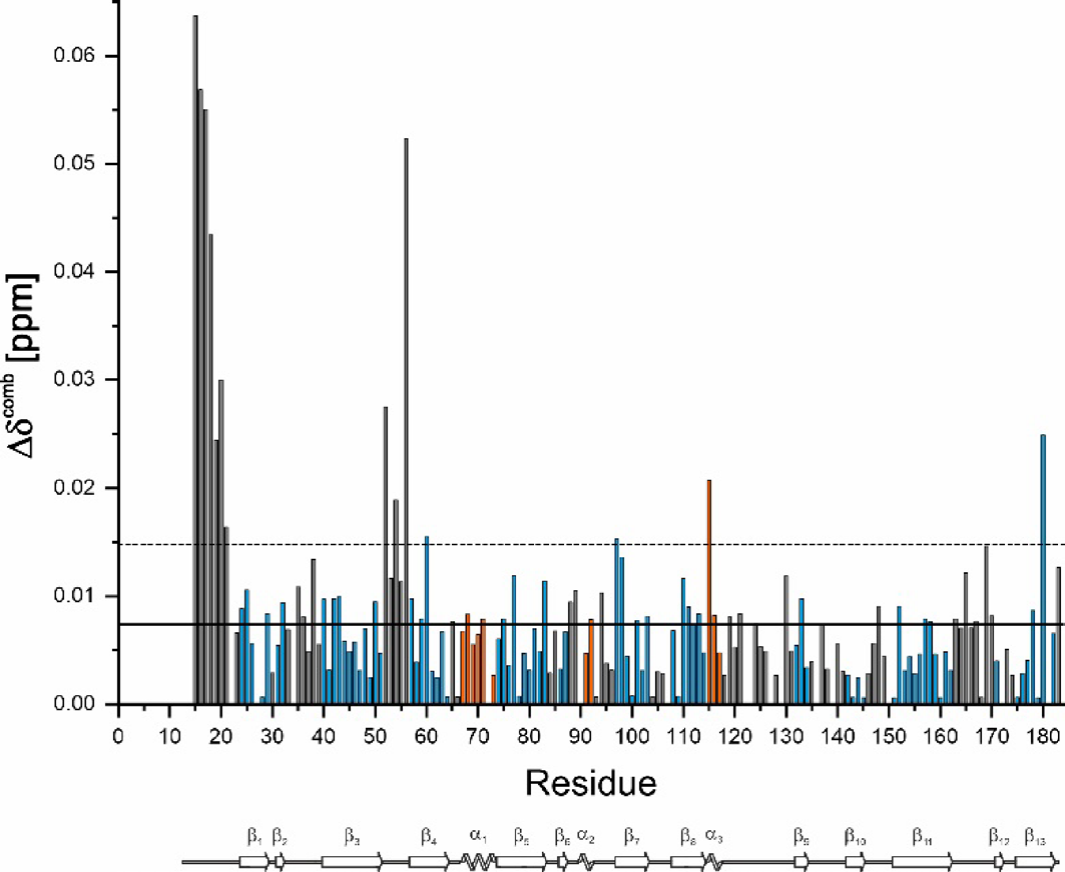
Chemical shift differences induced by N-terminal truncation. The combined ^1^H and ^15^N chemical shift changes Δδ^comb^^17^ are plotted as a function of the position in the amino acid sequence. Data were taken from the spectra shown in Fig. 10. Solid line, σ_0_^corr^ = 0.007 ppm; dashed lines: 2 σ_0_^corr^. β-pleated sheets are colored in blue, α-helices in orange. In addition, the secondary structure elements are depicted schematically.

Significant chemical shift differences > σ_0_^corr^ between monomeric CanA and K_1_-CanA are observed in the disordered region close to the point of truncation (T15, A16, Q17, A18, V19, S20, E21), in the loop in front of β4 (K52, Q54, N56) as well as in β4 of sheet B3 itself (L60), in helix α3 (K115), and in sheet B1 (β7, E97; β13, L180) (Fig. 11). These chemical shift differences may be caused by a transient, direct interaction of the N-terminus with these residues or by local conformational changes induced by truncation itself (see Discussion).

At a somewhat lower temperature of 313 K, two sets of resonances in the N-terminus of CanA could be identified in the three-dimensional spectra, suggesting conformational heterogeneity with at least two states. In addition, at this temperature also the resonances of the amino acids T2 to S5 are visible in the HMQC spectrum and can be assigned (Table 1). The second conformer is also characterized by a second set of C^α^ and C^β^-resonances (data not shown). At 323 K, the splitting of the S5, L7, F10, Y11, and A12 resonances is not observed anymore. From the chemical shift differences of the resonances, an upper limit of the exchange rate *k*_ex_ between the two states can be determined. Using the chemical shift difference of the amide ^15^N resonance of F10, an upper limit of the rate of 32.7 s^-1^ is obtained at 313 K.

### Binding sites for divalent ions of CanA and K_1_-CanA

**Fig. 12:**
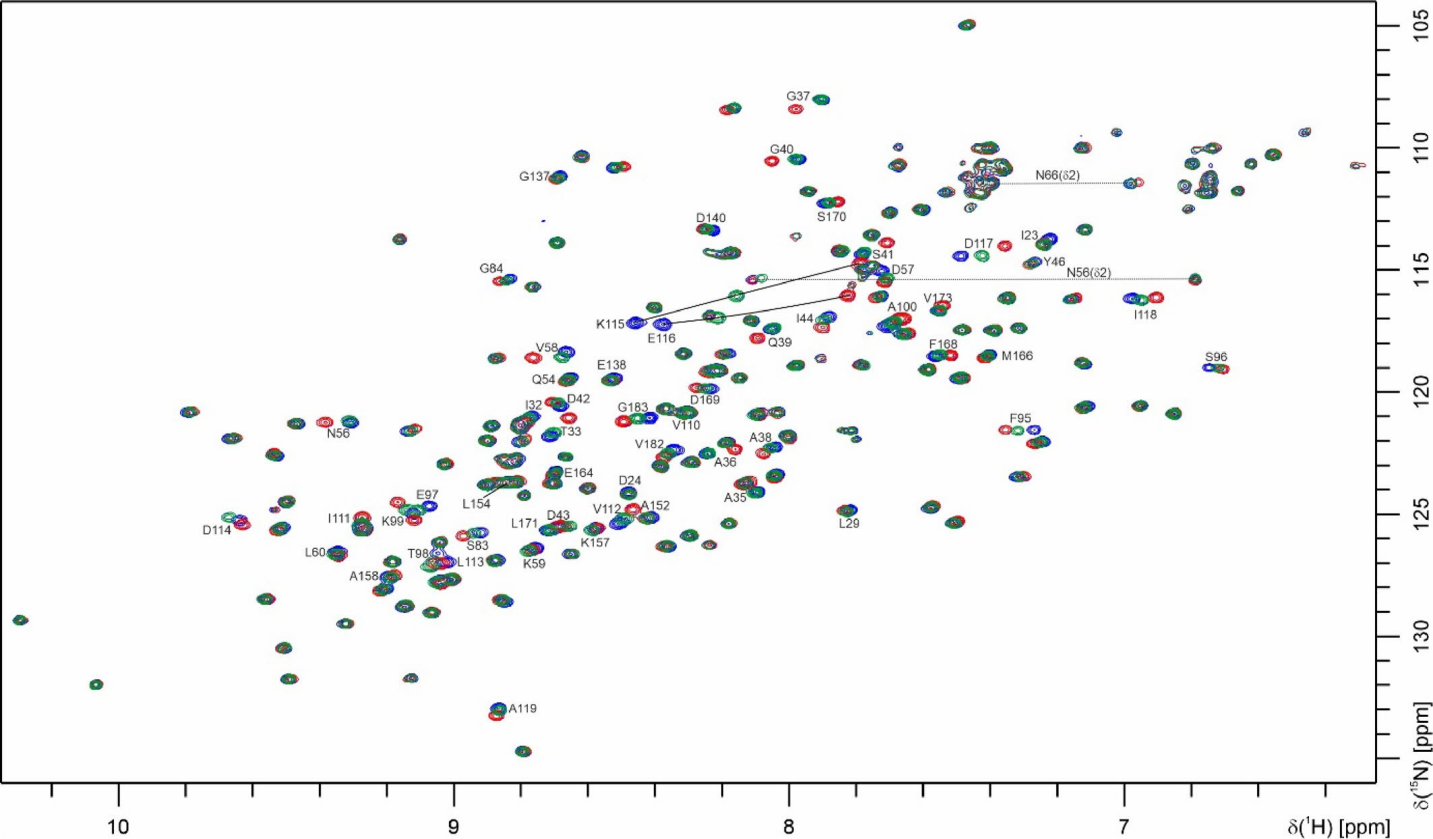
Ca^2+^-binding and Mg^2+^-binding to K_1_-CanA. [^1^H-^15^N]-SOFAST-HMQC of 0.12 mM ^15^N-enriched K_1_-CanA in 50 mM TRIS/HCl, pH = 6.6, 80 mM NaCl, 10% D_2_O, 0.4 mM DSS. Temperature 323 K, ^1^H-resonance frequency 800 MHz. (Blue) Reference spectrum; (red) CaCl_2_ added to an end concentration of 6.4 mM; (green) MgCl_2_ added to an end concentration of 6.4 mM.

The polymerization of CanA is induced by addition of the divalent ions Mg^2+^ and Ca^2+^. Since the full-length protein only weakly polymerizes in a buffer containing 50 mM Tris–HCl and 80 mM NaCl, the polymerization is not just an effect of ionic strength but requires specific interactions. The binding of small ligands can be indirectly detected and quantified by chemical shift changes induced by the addition of these ligands in 2D-HMQC spectra. A problem with CanA itself is that the binding of metal ions leads to polymerization and the disappearance of the NMR signals (see above) with time. A solution is the use of the non-polymerizing N-terminally truncated mutant K_1_-CanA. As an example, Fig. 12 shows the effect of the addition of CaCl_2_ or MgCl_2_ to K_1_-CanA in a [^1^H,^15^N]-SOFAST-HMQC spectrum. For the full analysis of the interaction, the CaCl_2_ concentration was varied in the range between 0 and 25.6 mM (corresponding to approximately twice its concentration in seawater). The spectrum shown in Fig. 12 corresponds to a CaCl_2_ concentration of 6.4 mM (molar ratio protein: CaCl_2_ of 1:53). Numerous peaks are shifted. They can be assigned during the titration by continuity of shifts. The largest effects are observed for K115 and E116. An analogous titration was performed with MgCl_2_ (Fig. 12). The MgCl_2_ concentration varied in the range between 0 and 51.2 mM (close to the magnesium concentration of seawater). The actual spectrum shown again corresponds to a concentration of 6.4 mM. The spectral effects of MgCl_2_ are quite similar to those of CaCl_2_ but substantially weaker.

**Fig. 13:**
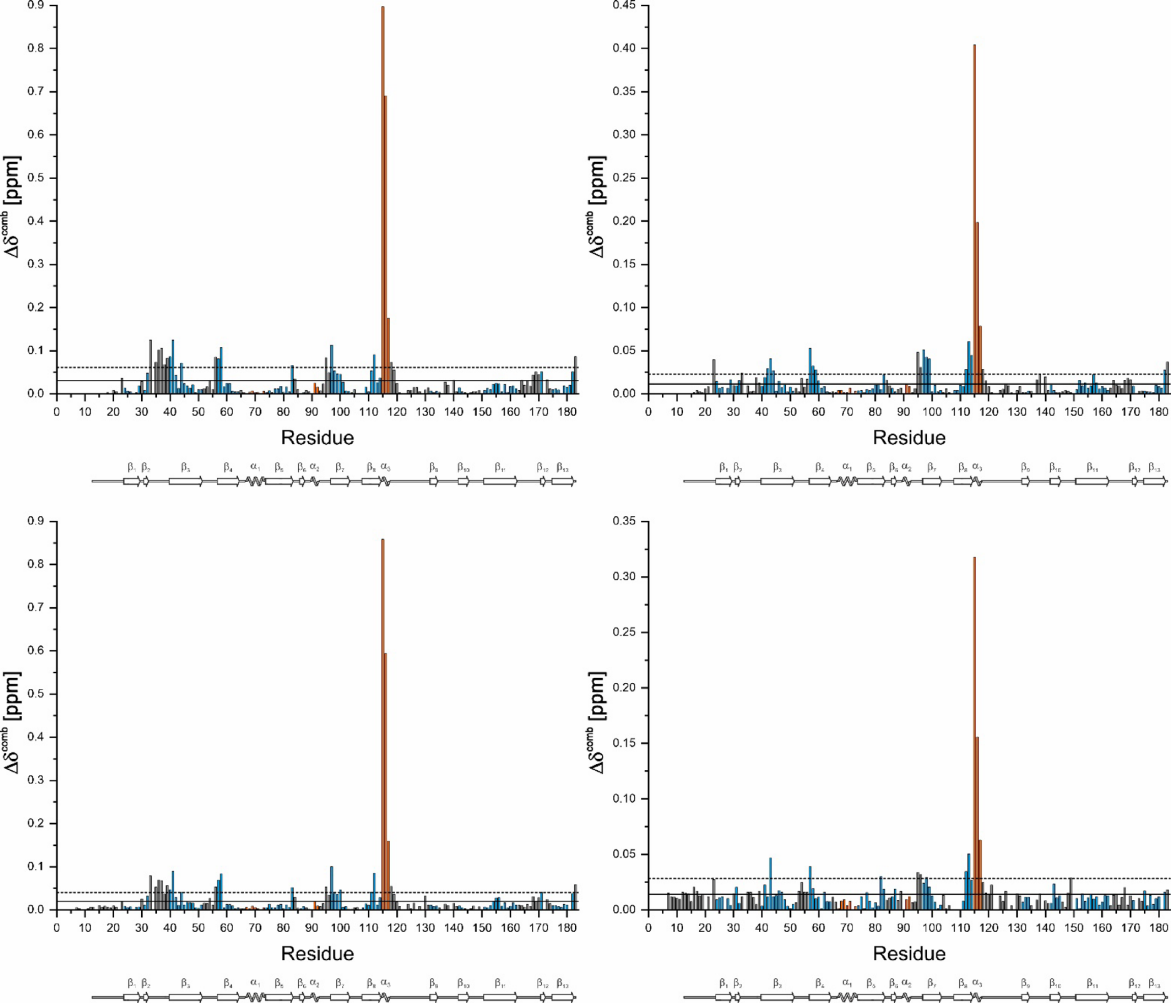
Identification of binding sites for divalent ions in CanA and K_1_-CanA by chemical shift perturbation. The combined ^1^H and ^15^N chemical shift changes Δδ^comb ^^17^ are plotted as a function of the position in the amino acid sequence. Residues with Δδ^comb^ > 2 σ_0_^corr^ likely are part of the binding site. K_1_-CanA data were taken from the spectra shown in Fig. 12. (Top) Titration of K_1_-CanA with CaCl_2_ (left) and MgCl_2_ (right), respectively. (Bottom) Titration of full-length CanA with CaCl_2_ (left) and MgCl_2_ (right), respectively. Solid line, σ_0_^corr^, broken line 2 σ_0_^corr^. β-pleated sheets are colored in blue, α-helices in orange. In addition, the secondary structure elements are depicted schematically.

For defining the binding sites, the atom and amino acid-specific combined chemical shift changes Δδ^comb^^17^, were plotted for K_1_-CanA and CanA as a function of the position in the sequence (Fig. 13). Note that the vertical scale for binding of Mg^2+^- ions is scaled up in the plot since the Mg^2+^- effects are generally smaller than those observed for Ca^2+^-ions. By far the largest Δδ^comb^-values are observed for K115 and E116 in the presence of Ca^2+^-ions as well as in the presence of Mg^2+^-ions. The general location of binding sites (more precisely, the residues where local structural changes are induced by ion-binding) can be predicted by changes of the combined chemical Δδ^comb^ in the presence of the ligands (Ca^2+^- or Mg^2+^-ions). In case that more than one ligand binds to a protein, the differences in NMR-derived affinities can be used to differentiate between different sites (see below). However, long-range structural changes may also lead to erroneous definitions of binding sites. Values above 2 σ_0_^corr^ are assumed to indicate direct interactions, whereas values in the range σ_0_^corr^ < Δδ^comb^ < 2 σ_0_^corr^ are often found in the second layer below the ligand binding sites themselves (Schumann et al., 2007).

The residues with significant chemical shift changes in the presence of Mg^2+^- or Ca^2+^-ions are listed for K_1_-CanA in Table 2. After the addition of MgCl_2,_ chemical shift changes are observed at similar positions as for CaCl_2_. The residues influenced by Mg^2+^- and Ca^2+^- binding are similar but not identical. From the 42 amino acids showing significant chemical shift changes in the presence of Ca^2+^-ions, only 9 do not show a significant Mg^2+^-effect. However, all negatively charged residues potentially involved in Ca^2+^-binding are also sensitive to the presence of Mg^2+^ ions. Of the 47 amino acids showing a significant chemical shift change in the presence of Mg^2+^-ions, 14 are not significantly influenced by the presence of Ca^2+^-ions.

**Table 2:**
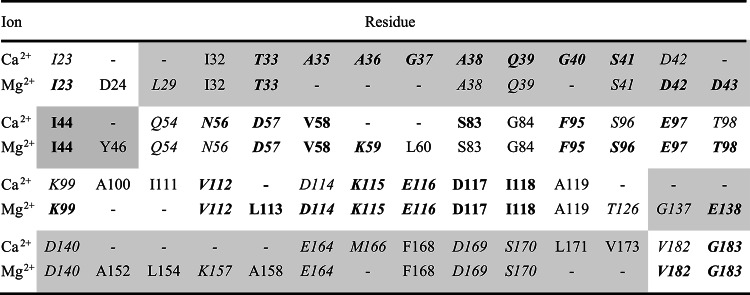
Residues shifting significantly after the addition of MgCl_2_ or CaCl_2_ to K_1_-CanA^a^.

^a^Residues with significant shifts Δδ > σ_0_^corr^, bold letters, Δδ > 2 σ_0_^corr^. Cursive letters, residues are surface accessible according to analysis with MolMol. Non-shaded and shaded areas, residues assigned to the binding areas BA1 and BA2, respectively. For quantitative shift values, see Fig. 13.

The chemical shift perturbation (CSP) induced by Ca^2+^- as well as Mg^2+^-binding produces very similar patterns in full-length CanA as already described in K_1_-CanA. However, the quality of the CanA CSP data is reduced since the quality of the SOFAST-HMQC spectra of full-length CanA in the presence of divalent ions is reduced. The general interacting pattern appears not to be perturbed by the presence and absence of the N-terminus. However, the affinities for divalent ions seem to be reduced in CanA in binding area BA1 and enhanced in binding area BA2 compared to K_1_-CanA (see Fig. 13).

**Fig. 14:**
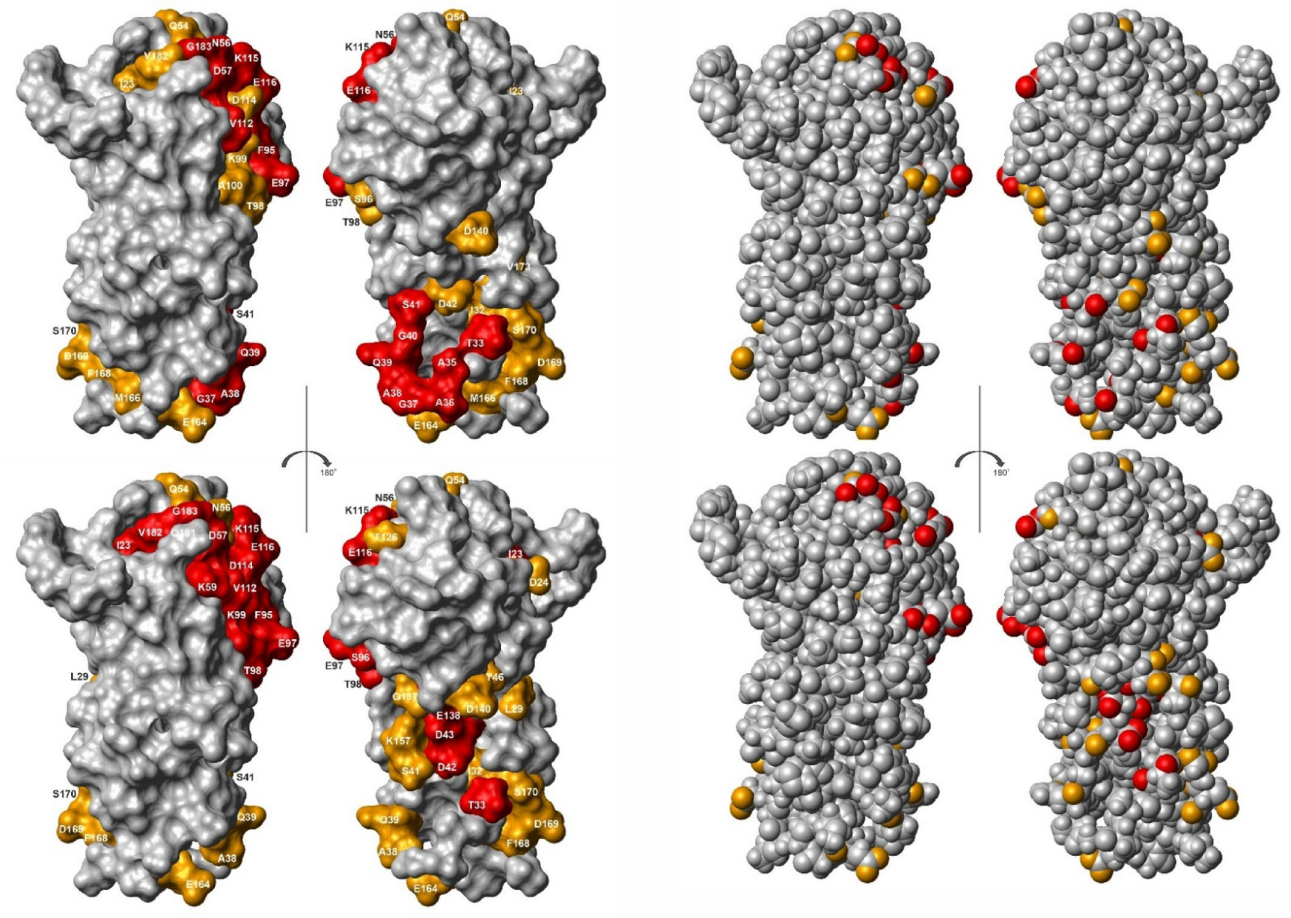
Binding sites of K_1_-CanA for divalent ions. Residues showing chemical shift changes Δδ^comb^ with (top) Ca^2+^-binding and with (bottom) Mg^2+^-binding. (Left two panels) residues with σ_0_^corr^ < Δδ^comb^ < 2 σ_0_^corr^ (red), residues of with Δδ^comb^ > 2 σ_0_^corr^ (orange). (Right two panels) presentation of oxygen atoms of the significant residues on the protein surface. Structures were represented by MolMol^15^.

The residues influenced by binding of divalent ions are represented on the surface of the K_1_-CanA structure in Fig. 14. The chemical shift analysis also identifies residues that can only be indirectly influenced by ion binding, such as residues in the second layer, since only residues on the surface can directly interact with the divalent ions (Table 2). With this restriction, two spatially separated binding areas for positively charged divalent ions BA1 and BA2 can be defined. The putative Ca^2+^ -binding area BA1 contains the five negatively charged and solvent accessible residues D57, E97, D114, E116, and G183 (with the negatively charged C-terminal carboxyl group). The putative Ca^2+^-binding area BA2 contains the four negatively charged residues D42, D140, E164, and D169. For Mg^2+^-binding, small differences are observed.

For full-length CanA (in a concentration 0.12 mM), a set of [^1^H-^15^N]-SOFAST-HMQC spectra in the absence and presence of 0.6 mM CaCl_2_ and a set of [^1^H-^15^N]-SOFAST-HMQC spectra in the absence and presence of 1.2 mM MgCl_2_ have been recorded. The polymerization process after the addition of divalent ions is slow enough for recording the sets of SOFAST-HMQC spectra with sufficient quality (total recording time per spectrum 51 min). The additional N-terminal amino acids do not change the general CSP pattern of ion binding much. The CaCl_2_ and MgCl_2_-induced chemical shift patterns are very similar to truncated CanA. However, some differences are observable (see Fig. 13). In the N-terminal segment, missing in K_1_-CanA, a significant specific interaction with divalent ions could not be observed for Ca^2+^ ions, but for Mg^2+^ ions (L7, A12, T13, G14, A16, Q17, V19).

### Affinity of CanA and K_1_-CanA for Ca^2+^- and Mg^2+^-ion

By addition of suitable quantities of CaCl_2_ or MgCl_2,_ the affinities for these ions can be determined by recording the HSQC-spectra of the protein and fitting the ligand-induced combined chemical shift changes Δδ^comb^. However, the CSP method does not detect the direct interaction of the divalent ions with a specific atom of a residue, but local (and eventually global) small conformational changes induced by binding of these ions. The exact binding site of a ligand inducing significant ^1^H and ^15^N backbone shifts of a given residue is primarily unknown, but in most cases is close to that residue. Since we have a larger number of residues distributed over the surface of the protein (Fig. 14), several divalent ions must bind simultaneously to our protein. Therefore, in principle, the data must be fitted assuming *j* binding sites with *N*_*i*_-ligands bound to each site. Every binding site may have a different microscopic dissociation constant *K*_D_^j^. Different types of binding may occur simultaneously as independent and cooperative binding (see e.g.^18^). From the concentration dependence of chemical shifts, the absolute number of the binding sites *i* cannot be determined. Therefore, the *N*_*i*_ were set arbitrarily to 1 for the calculations based on Eq. 1. The number of binding sites themselves can be partly derived from their location in the three-dimensional structure by assuming local interactions. The minimum number of interaction sites can be derived from significant differences in the corresponding affinities.

**Fig. 15:**
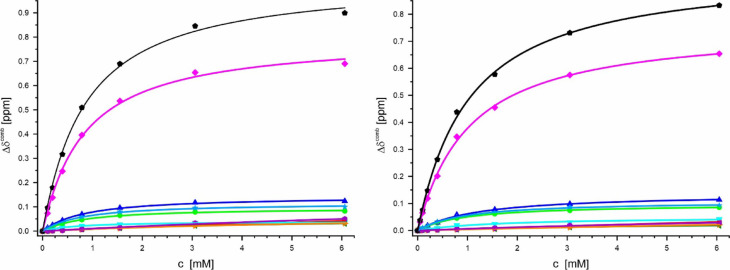
Ca^2+^-binding to K_1_-CanA and CanA. (Left) K_1_-CanA, (right) CanA. The changes Δδ^comb^ of the amide groups are plotted as a function of the total CaCl_2_ concentration. () D42 (24 mM; 50 mM), () D57 (0.74 mM; 0.88 mM), () E97 (0.74 mM; 1.1 mM), () D114 (0.58 mM; 1.6 mM), () E116 (0.73 mM; 0.94 mM), () D140 (4.5 mM; 7 mM), () E164 (7 mM; 28 mM), () D169 (18 mM; 12 mM), () K99 (0.69 mM; 0.88 mM), () K115 (0.74 mM; 0.95 mM). Values in brackets are dissociation constants *K*_D_ calculated for each amino acid individually for K_1_-CanA and CanA, respectively.

Figure 15 shows the combined ^1^H and ^15^N chemical shift dependence of the amide groups of selected amino acids for binding of Ca^2+^-ions to K_1_-CanA and full-length CanA. Binding of Ca^2+^- and Mg^2+^- ions usually requires the interaction with negatively charged and polar groups (see Discussion). Both proteins contain 21 negatively charged residues (Asp and Glu) and 12 positively charged lysines (and one histidine, no arginine). The negatively charged and polar groups of those residues that show significant shift changes in the presence of divalent ions and are located on the surface of the protein are depicted in Fig. 14. In Fig. 15, the corresponding fit curves for the 8 negatively charged residues located on the surface of the proteins with amide resonances shifting significantly with addition of CaCl_2_ are depicted. In addition, the fit curves of two lysine residues with amide resonances shifting significantly in the presence of divalent ions are shown. For a more complete characterization of the binding sites and the calculation of the corresponding dissociation constants, all amino acids with CaCl_2_-induced shifts above σ_0_^corr^ were selected. Residues where no reliable fits of the experimental data were obtained were excluded from the final analysis. Clearly, at least four sets of binding constants can be detected. Together with the spatial analysis, most probably 5 different binding sites for divalent ions are needed to explain the data. Qualitatively, the analysis of binding curves of K_1_-CanA and CanA gives very similar results for the two proteins. During the experiments with CanA, a slow polymerization occurs after the addition of CaCl_2,_ which increases with higher CaCl_2_ concentrations. This leads to decreased signal intensities with time, but the shift changes can still be followed in the whole concentration range.

**Fig. 16:**
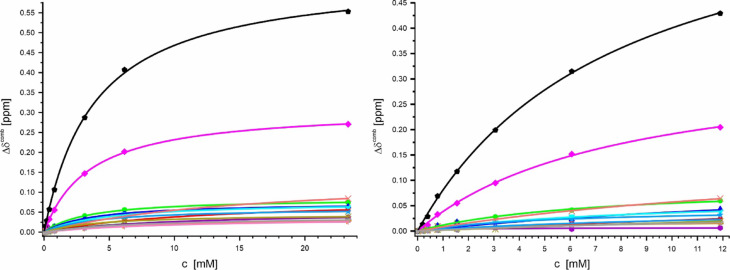
Mg^2+^-binding to K_1_-CanA and CanA. (Left) K_1_-CanA, (right) CanA. The changes Δδ^comb^ of the amide groups are plotted as a function of the total MgCl_2_ concentration. () D24 (10.3 mM; 12 mM), () D42 (12 mM; 40 mM), () D43 (15.4 mM; 18 mM), () D57 (3.3 mM; 6.8 mM), () E97 (3.6 mM; -), () D114 (4.6 mM; 9 mM), () E116 (3.36 mM; 7.7 mM), () D140 (9.3 mM; 23 mM), () E164 (8.0 mM; 6.4 mM), () D169 (11.1 mM; 1), () K59 (4.1 mM; -), () K99 (4.0 mM; 5.3 mM), () K115 (3.7 mM; 7.7 mM), () K157 (7.7 mM; 15 mM). Values in brackets are dissociation constants *K*_D_ calculated for each amino acid individually for K_1_-CanA and CanA, respectively.

The same experiments were performed for the two proteins with MgCl_2_. The obtained binding curves are depicted in Fig. 16. As in the case of titration with CaCl_2_, CanA starts to polymerize by the addition of MgCl_2_. Again, most of the apparent *K*_D_s are higher in CanA than in K_1_-CanA.

The apparent *K*_D_ values of different residues in the two binding areas are summarized in Table 3. They were calculated under the assumption of independent binding to the residue under question. In BA1 for all residues studied, very similar Ca^2+^-binding constants are obtained (Fig. 15). However, they significantly differ in the magnesium affinity. A special case in BA1 is D114 with a significantly lower *K*_D_ for Ca^2+^ compared to the other residues of this region. Since the *K*_D_-values of binding area BA1 for Ca^2+^-ions are much smaller than those for Mg^2+^ ions in CanA and K_1_-CanA, one can assume that these sites are Ca^2+^-binding sites. In the binding area BA2, the obtained affinities are substantially lower than those in BA1. In addition, the differences in the apparent *K*_D_-values for Ca^2+^- and Mg^2+^-ions are much smaller, indicating that these interaction sites have a low specificity for the type of divalent ions.

**Table 3:** Apparent dissociation constants of K_1_-CanA and CanA for divalent ions^a^.

Protein		K_1_-CanA	CanA	K_1_-CanA	CanA
Ion		Ca^2+^	Ca^2+^	Mg^2+^	Mg^2+^
Binding site	Residue	*K*_D_ [mM]			
BA1	D57	0.74 ± 0.05	0.88 ± 0.04	3.3 ± 0.1	6.8 ± 0.9
	E97	0.74 ± 0.05	1.1 ± 0.01	3.6 ± 0.1	-^c^
	K99	0.69 ± 0.06	0.88 ± 0.06	4.0 ± 0.2	5.3 ± 0.6
	D114	0.58 ± 0.04	1.6 ± 0.3	4.6 ± 0.5	9 ± 1
	K115	0.74 ± 0.04	0.95 ± 0.04	3.7 ± 0.1	7.7 ± 0.4
	E116	0.73 ± 0.05	0.94 ± 0.04	3.36 ± 0.06	7.7 ± 0.5
	G183	0.90 ± 0.04	1.03 ± 0.03	5.0 ± 0.24	8 ± 3
BA2	D42	24 ± 9	-^c^	12 ± 1	39 ± 21^b^
	D140	4.5 ± 0.5	7 ± 2^b^	9.3 ± 0.7	23 ± 6
	E164	7 ± 1	28 ± 6^b^	8.0 ± 0.6	6.4 ± 0.7
	D169	18 ± 9	12 ± 3^b^	11.1 ± 0.8	1.2 ± 0.9

^a^0.17 mM ^15^N-enriched K_1_-CanA or 0.12 mM ^15^N-enriched CanA in 50 mM Tris/HCl, pH = 6.6, 80 mM NaCl, 10% D_2_O, 0.4 mM DSS. Temperature 323 K. The *K*_D_-values were calculated for charged residues in the areas with Δδ^comb^-values above σ_0_ in K_1_-CanA and/or CanA, respectively. The *K*_D_-values are apparent *K*_D_-values since the number *N*_*i*_ of ligands bound to site *i* is not known. Data were fitted individually assuming *N*_*i*_ = 1 with Eq. 1. Values are the averages of the combined ^1^H, ^15^N chemical shift changes at a given site.

^b^Shifts not significant in CanA.

^c^No stable fit obtained.

Positively charged divalent ions should mainly interact with negatively charged amino acids. However, the amino acids close to these residues also sense the binding of the positively charged divalent ions. Especially large effects were observed for K99 and K115 with apparent *K*_D_-values close to those of the adjacent negatively charged amino acids (E97, D114, E116).

## Discussion

### Three-dimensional structure of K_1_-CanA and CanA

We have determined the solution structure of K_1_-CanA, the N-terminally truncated form of CanA. The comparison of the CD spectra (Fig. 2, right) as well as the two-dimensional NMR spectra of CanA and K_1_-CanA (Figs.10, 11) shows only small spectral changes, indicating that the general three-dimensional structure is preserved in the truncated form. Except for other cannulae-forming proteins of *Pyrodictium abyssi* itself, CanA does not have significant sequence homologies to other proteins. In the genome sequence of *Pyrodictium occultum* possible genes of cannulae proteins were identified^19^.

The basic fold of CanA represents a non-canonical jellyroll class I. The main fold consists of two large β-pleated sheets B1 (β_1_, β_3_, β_5_, β_7_, β_11_) and B3 (β_14_, β_8_, β_13_), forming the jellyroll, and the α-helix α_1_. It is completed by two additional smaller β-pleated sheets B2 (β_2_, β_12_) and B4 (β_6_, β_9_, β_10_), and the two helices α_2_ and α_3_. The one-turn helix α_3_ is predicted by the analysis of the backbone chemical shifts, but not formed perfectly in all NMR structures. The β-sheets, B2 (β_2_, β_12_) and B4 (β_6_, β_9_, β_10_), do not belong to the jellyroll.

The structure contains 12 lysine residues, 8 of which are at a distance from an aspartate or a glutamate residue that would allow the formation of salt bridges (side chain N–O distance < 0.4 nm)^20^. Therefore, salt bridges K52-D53, K59-D57, K71-E68, K75-E164, K99-D117, K115-E116, K125-D124, and K141-D138 could be formed. The formation of salt bridges is assumed to be essential for creating high-temperature stability^21^. In some of the NMR structures, K99 (in β-sheet β_7_) forms an internal salt bridge to D117 (in helix α_3_), and K115 forms a salt bridge with E116 (both in putative helix α_3_).

The first 10 (11 when including the N-terminal methionine) amino acids of the N-terminus of CanA have been removed in K_1_-CanA. Their removal induces highly significant, local chemical shift changes (Figs. 10 and 11). This includes amino acids 15 to 21 of the unstructured N-terminus of K_1_-CanA but also K52, Q54, N56, L60, E97, K115, and L180, located in the well-folded part of the structure, the ion binding area BA1. A plausible hypothesis would state that it is an effect of a transient interaction of the N-terminal amino acids with this area in the solution state. In line with this, we observe a chemical shift heterogeneity (two states in slow exchange) of the amino acids S5, L7, F10, Y11, and A12 (Table 1). However, the spectroscopic data show that the global fold of the protein monomer is not influenced by the removal of the first 10 (11) N-terminal residues (Fig. 2, right).

### Comparison of the solution structure of CanA with its structure in the cannulae

**Fig. 17:**
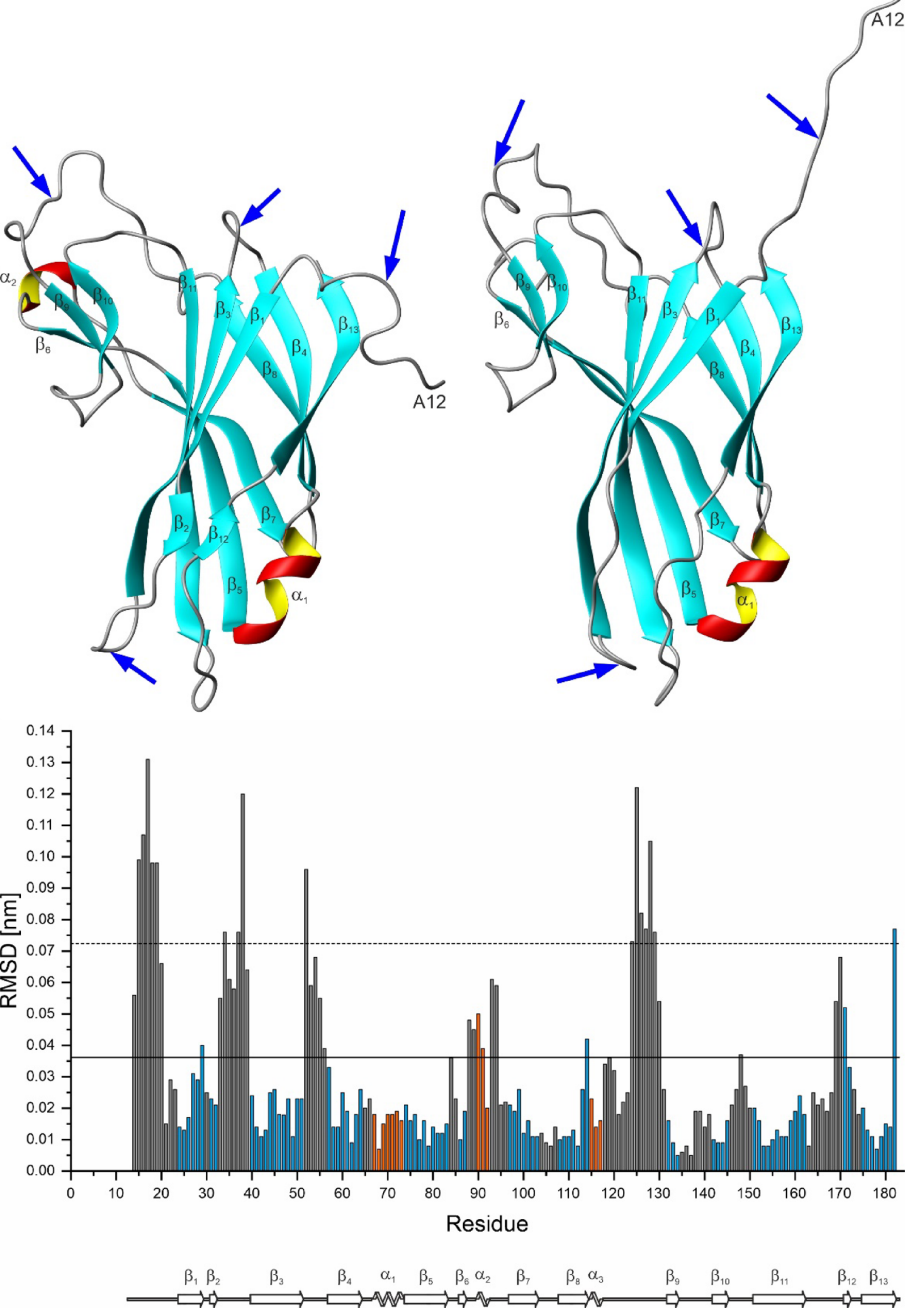
Structural differences between free CanA in solution and CanA as part of the cannulae. (Top left) NMR structure of K_1_-CanA (this paper), (top right) EM-structure of CanA (PDB accession # 7UII). Arrows show regions with highly significant structural differences. (Bottom) Sequence-dependent local RMSDs of the two structures. Values above the reduced standard deviation to zero σ_0_ (solid line) are considered significant, and those above 2 σ_0_ (broken line) as highly significant. β-pleated sheets are colored in blue, α-helices in orange. In addition, the secondary structure elements are depicted schematically. Structures were represented by MolMol^15^.

During the preparation of this paper, the EM structure of polymerized CanA was made available in the PDB database with literature status “to be published” (PDB accession # 7UII). The structure of CanA in solution and the structure of CanA embedded in the cannulae are quite similar. The main chain RMSD (N, C^α^, C′) of the well-folded parts of two proteins (amino acids 22 to 183) is only 0.239 nm. Both proteins show a jellyroll class I fold (Fig. 17). The structural differences required for polymerization can be derived from a comparison between the two structures (neglecting possible errors from EM-reconstruction with a relatively low resolution compared to the NMR structure). The local differences can be quantified by calculating the local RMSD differences in the protein backbone (Fig. 17). As expected, they mainly focus on the protein–protein interaction sites.

Large differences are observed for the unfolded N-terminus of CanA, which has a largely different conformation in full-length and truncated CanA. As NMR spectroscopy shows (Table 1), in monomeric state the N-terminus of CanA exists in two different conformations in slow exchange with an upper limit of the exchange rate *k*_ex_ of 31.7 s^-1^ at 313 K. Since the removal of the 11 N-terminal amino acids leads also to significant spectral changes of the NMR spectra (Figs. 10, 11), a plausible hypothesis is that one of the two states is a state where the N-terminal segment is in direct contact with the main body of the protein monomer in solution (K52, Q54, N56, L60, E97, K115, and L180). In this state, the N-terminal segment could block a possible binding site required for proper polymerization. In contrast, in the polymer structure, the N-terminus is extended and in close contact with other protomers. In addition, the small β-pleated sheet B2 and the α-helix α_2_ do not exist in the cannula structures solved by cryo-EM. The largest local RMS differences are observable in the loop regions connecting the secondary structure elements, i. e. the loops between β_2_ and β_3_, β_3_ and β_4_, β_6_ and α_2_, between α_3_ and β_9_, and between β_11_ and β_12_ (highlighted by arrows in Fig. 17). These regions also contain the majority of the binding sites for divalent ions.

**Fig. 18:**
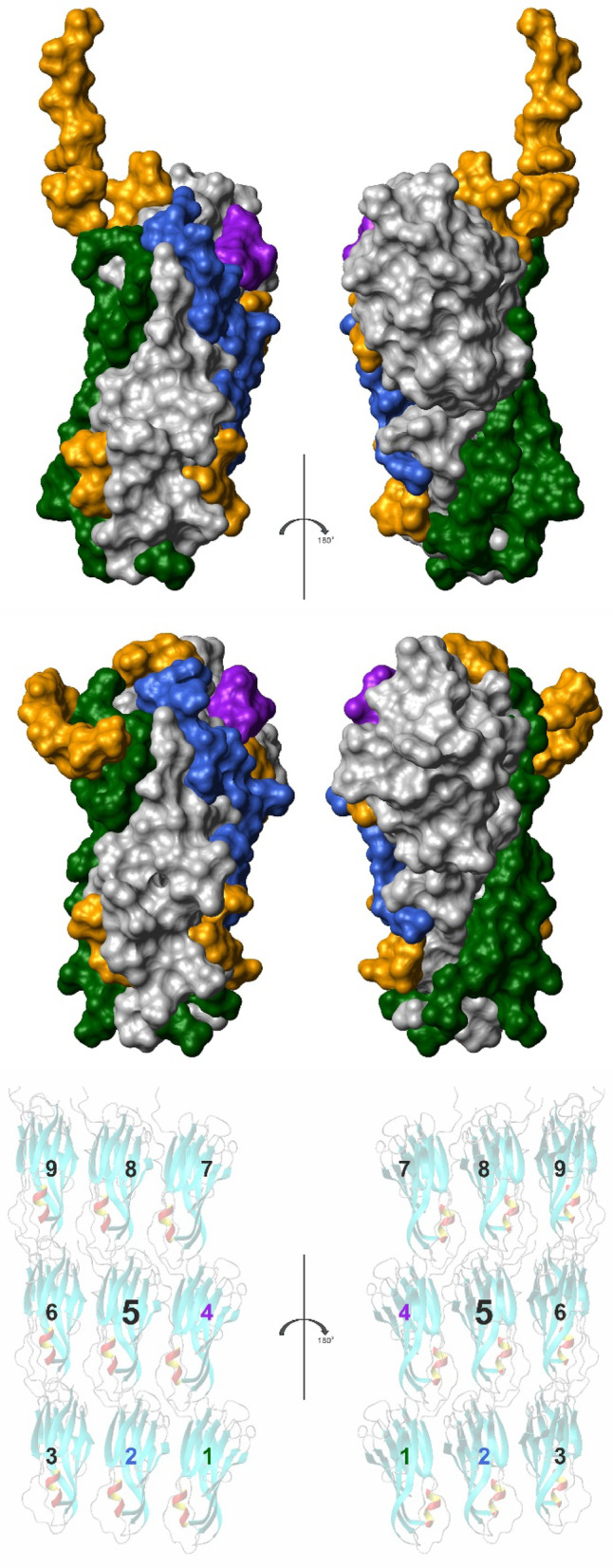
Protein–protein interaction in cannulae. Residues interacting in the cannulae structure 7UII with neighboring protomers are highlighted in the cryo-EM structure (top) and in the equivalent solution NMR K_1_-CanA structure (middle). Hydrogen atoms were added in the cryo-EM structure. Only residues 12 to 183 are represented. The residues interacting with the N-terminal residues 1-22 of the neighboring protomers in 7UII, relative to central protomer 5, are represented in blue (protomer 1), green (protomer 2), and purple (protomer 4), respectively. The remaining interaction surface is highlighted in orange. Orientation of the K_1_-CanA structure as viewed from the interior (left) and the exterior (right) of the cannulae as in Figs. 4 and 14, and identical to protomer 5 in the representation (bottom). Structures were represented by MolMol^15^, and interacting residues were defined as described in^17^.

The structural differences most probably represent structural adaptations that are necessary for the formation of the polymer contacts. Each CanA subunit has interactions with 8 direct neighbors. The N-terminus itself is not in contact with the main body of its protomer. In contrast, it now stabilizes the formation of two layers of the helical arrangement. The interacting residues of the central protomer are depicted in Fig. 18. The contacting residues were defined according to^17^ based on the change of the accessible surface in the multimer and interatomic distances. They were either plotted on the surface of the cryo-EM structure (Fig. 18, top) or the NMR-structure (Fig. 18, middle). For a better comparison with other data, the protomers were represented in the same orientations used in Figs. 4 and 14, and identical to the central protomer in the representation (Fig. 18, bottom). As already suggested, the ion binding sites are the main interacting points in the complex. Somewhat surprisingly, a large part of the contacts are formed by the N-terminal segments and also encompass areas at the inside and outside of the cannulae.

In Figs. 4 and 14, the structures are presented in such a way that the left panel shows the surface of the molecule oriented towards the inside of the cannula and the right panel shows the surface oriented towards the outside. The two ends of the β-pleated sheets forming the jellyroll contain large loops that could adapt to the three top and the three bottom neighboring molecules. Here, an induced fit process could occur. However, these relatively large reorientations are probably induced by ion binding and are probably required by a conformational selection mechanism (at least two conformational states of the molecule).

### Structural variations between different Can-proteins

CanA has a large jellyroll class I fold structure consisting of two β-pleated sheets B1 and B3, complemented with the smaller β-pleated sheets B2 and B4 and a helix α_1_. The β-pleated sheet B2 and the small helix α_2_ form a well-separated additional domain (Figs. 3 and 17). This additional domain is oriented towards the outside of the cannula and is missing in CanB and CanC. Its presence may hinder a bifurcation of the cannulae that is not observed in the homogeneous cannulae produced by recombinant CanA only, but is observed in cannulae from natural sources containing also CanB and CanC. The β-sheets B2 and B4 do not belong to the jellyroll and are probably also important for the correct formation of cannulae.

### Polymerization reaction in the absence and presence of divalent ions

The polymerization reaction of CanA induced by divalent ions is quite slow with a time scale of hours (Fig. 7). The experiments show that the polymerization from the monomeric state has at least two different phases with time constants separated by almost one order of magnitude. It is tempting to identify the two phases with a primary oligomerization of the monomers (fast) and an arrangement of the cannulae structure (slow). The obtained CanA polymers (cannulae) are very stable even at 401 K. This holds for the biosynthetically obtained protein as well as the protein obtained from natural sources. Cannulae are stable at temperatures higher than the boiling point of water at ambient pressure (373 K), a fact that reflects the high depth at which the archaeal cells are found (depth of 3600 m below sea level). Even in the absence of divalent ions, some cannulae are formed in thermal equilibrium and coexist in low relative concentration with monomers and oligomers in solution (Fig. 1, middle). However, such an equilibrium should be expected as long as the total CanA concentration is higher than the critical concentration for polymer formation that depends on the experimental conditions (e. g. temperature, pressure, concentration of divalent ions). NMR diffusion and dynamic light scattering measurements estimate a hydrodynamic radius of CanA of 1.68 nm and 1.74 nm at pH 6.6 and 318 K indicating that freshly dissolved CanA after gel filtration exists predominantly as a monomer at this pH. This is different at pH 9.0 where an apparent molecular mass of 64.63 kg/mol was determined, that would approximately correspond to a trimer in a homogeneous solution (monomer 19.85 kg/mol) or more probably the coexistence of monomers with larger polymers. This trimer may represent the first characteristic oligomer before polymerization. However, even this is a slow process since the polymers can be separated by gel filtration from the monomers before they reform again. Since usually pH-switches are caused by protonation/deprotonation of charged amino acids in the questionable pH-range around pH 7, the most probable candidate for such a switch close to neutral pH is a histidine. Since CanA contains only a single histidine residue, H28 in β-strand β_1_ is the natural candidate.

In the presence of Mg^2+^- and/or Ca^2+^-ions at concentrations as they occur in seawater, the equilibrium is shifted from monomers or smaller oligomers almost completely towards the formation of high molecular mass polymers (cannulae) (Fig. 7). From the NMR data the critical concentration of CanA can be estimated that is required for polymerization. At 298 K and pH 7.5 in the presence of 20 mM MgCl_2_ and 20 mM CaCl_2_, the critical concentration is 2.48 μM. Note that, in first approximation, the critical concentration also corresponds to the *K*_D_ for the dissociation of monomers from the free ends of the polymer. As expected (and confirmed by our experiments), the critical concentration and the corresponding *K*_D_ is much higher in the absence of divalent ions. At the “physiological” conditions of 35 MPa and 373 K where *Pyrodictium abysii* is growing optimally, the critical concentration should be different, but it should be low enough to enable polymerization. Unfortunately, we cannot perform these high-pressure, high-temperature experiments for technical reasons.

After polymerization, some weak signals with line widths of the order of 20 Hz remain with chemical shifts typical for random-coil structures. They could correspond to highly mobile regions in the large polymers, which are characterized by low local motional correlation times. Such behavior was first reported for the C-terminus of rabbit skeletal muscle myosin^22^. Two resonances are easily assigned, namely the methyl resonances of M1 and M166. Therefore, two regions with local high mobility in parts of the polymer can be identified as the N-terminal amino acids and the loops connecting β-strands β_11_ and β_12_ of β-pleated sheets B1 and B2.

### Binding of divalent ions and related structural effects

Divalent ions induce the polymerization of full-length CanA. We have studied the effect of Mg^2+^- and Ca^2+^-ions on the HSQC- and HMQC spectra of ^15^N-enriched K_1_-CanA as well as full-length CanA. It turns out that there are two main regions of the protein that are influenced by the binding of divalent ions, binding areas BA1 and BA2 (Fig. 13). BA1 forms a compact region that is located close to the upper rim of the β-sheets forming the jellyroll at the inner side of the cannulae. The binding area BA2 is more diffuse and not as well defined as BA1. Contrary to BA1, it is located predominantly on the outer side of the cannulae. Assuming a stoichiometry of 1, the dissociation constant for Ca^2+^-ions at BA1 is about 0.7 mM and 1.1 mM in K_1_-CanA and CanA, respectively. The *K*_D_ for Mg^2+^-ions is significantly higher (Table 3). However, at the salt concentrations found in seawater (10.5 mM Ca^2+^, 54.0 mM Mg^2+^, IASPO reference seawater), these site(s) should be fully occupied by Ca^2+^- and/or Mg^2+^-ions. In BA2, the affinity for divalent ions is about an order of magnitude lower (Table 3). The Ca^2+^-affinity is not high enough to lead to a complete saturation of all sites. However, the Mg^2+^-affinity is still sufficient to grant complete binding at the Mg^2+^-concentrations of seawater. In this respect, for practical purposes they are Mg^2+^-binding sites.

Chemical shift mapping is a powerful method to study binding of small ligands on proteins. However, the direct magnetic or electric effects on the protein chemical shifts are usually rather small. The main chemical shift changes of the backbone atoms are assumed to be caused by small, local conformational changes that also may influence the second protein layer, not located on the surface of the protein (Schumann et al., 2007). However, it is a plausible assumption that the residues with the highest chemical shift changes after binding are probably involved in the direct interaction. In binding area BA1 five negatively charged residues, D57, E97, D114, E116, and G183 (with the negatively charged C-terminal carboxyl group) are present. Together with K99, K115, and D117 (the latter is not accessible from the surface), they have the largest chemical shift changes induced by Ca^2+^-interactions in the protein (21, 14.7, and 3.7 σ_0_) by far. In binding area BA2, again four negatively charged residues, D42, D140, E164, and E169, exist. They have significant but not excessive chemical shift changes with binding of divalent ions. Since they also are not characterized by similar *K*_D_ values, they cannot define a single binding site for divalent ions. The differences of affinity would require at least 4 binding sites of divalent ions (Table 2). Taking into account the different locations probably 4 to 5 binding sites for divalent ions exist (see Results). This is also in line with the observation that CanA (and K_1_-CanA) are strongly negatively charged. They contain 21 negatively charged residues (Asp and Glu) and 12 positively charged lysines (and one histidine, no arginine). This would make a surplus of 8 to 9 negative charges (depending on the protonation state of H28). Binding of 4 to 5 positively charged divalent ions would lead to electroneutrality of the protein.

Classical high-affinity Ca^2+^-binding sites (as in the EF-hands of calmodulin) are built by four negatively charged side chains with sequences such as DxDxDGxxxxxE or the Excalibur motif with the sequence DXDXDGXXCE^23,24^. The side chains of the aspartic acids form monodentate complexes with the Ca^2+^-ion, and the glutamate side chain forms a bidentate complex. However, these sequence motifs cannot be found in CanA. Even the shorter, general calcium-binding motif DxDxDG^25^ is not present in the structure. However, the affinity of the Ca^2+^ and Mg^2+^ sites required for the proper polymerization of CanA should have moderate affinities, since they should only become occupied at the high concentrations of divalent ions prevailing in the seawater where the polymerization should be induced.

### Ion binding and polymerization

Polymerization of CanA is induced by the binding of divalent ions at concentrations typical for seawater. Three mechanisms may enforce protein polymerization: (1) Induction of conformational changes of the monomeric units that are required for a perfect fit of the structure into the polymeric structure, (2) screening of charges in neighboring units that would inhibit polymer contacts, and (3) direct formation of intermolecular salt bridges. The specific chemical shift changes caused by binding of divalent ions in BA1 and BA2 are indicative of larger structural changes induced in the monomeric units. This would be in line with mechanism 1 and could support a conformational selection required for polymerization. In addition, the solution structure is quite different from the cryo-EM structure in the regions showing large chemical shift perturbations after ion binding (Fig. 17). The comparison of K_1_-CanA with full-length CanA suggests that in the monomer the N-terminal strand is in the thermal equilibrium in partial contact with the binding area BA1 with a strong contact to amino acids K115 and D116 (Fig. 11). Binding of a divalent ion in this region may weaken this contact. Correspondingly, the affinity for divalent ions in full-length CanA in BA1 should be decreased compared to that observed in truncated Can. This is indeed observed (Table 3). The removal of the N-terminal strand from BA1 would again favor the polymerization since the N-terminus is extended in the cannulae structure and contacts here two other CanA subunits. A neutralization of the negative charges concentrated in BA1 and BA2 by binding of positively charged divalent ions is probably necessary for the interaction of the uncharged N-terminal segments from two neighboring protomers that interact with these regions (see Figs. 17 and 18).

A preprint newly available during the revision of this manuscript^26^ present three Ca^2+^-binding sites in the cryo-EM structure of polymerized CanA that all interconnect two protomers. One Ca^2+^-ion is observed that binds to the side chains of D114 and E116 (connecting loop β_11_ and β_12_) of the central protomer and D’24 (first residue of β_1_) of a neighboring protomer. A second Ca^2+^-ion binds Q54 and N56 of the central protomer and to Q’39 and G’183 of a neighboring protomer. The third Ca^2+^-ion binds to V182 of the central protomer and to G’37, E’161, and E’164 of a neighboring protomer. These binding sites are not (and cannot be) completely preserved in the monomer structure. D24 (located in BA1) shows only significant shift changes in the presence of Mg^2+^-ions, and G37 (BA2) in the presence of Ca^2+^-ions. Q39 and E164 located in BA2, Q54, N56, D114, E116, V182, and G183 located in BA1 show significant shift changes in the presence of both divalent ions. E161 does not show significant chemical shift changes in the presence of divalent ions in the monomer. This supports the model that binding of divalent ions to the isolated monomer mainly induces the conformational changes required for polymerization. In the next step, the ion binding itself has to reorganize to bridge different protomers of the polymer. Note that we can now estimate the affinity of these groups in the monomer. D114 has a Ca^2+^-dissociation constant of 0.58 mM, E116 of 0.73 mM, E164 of 7 mM, and G183 of 0.9 mM in K_1_-CanA. The affinity of D114 is strongly perturbed by the presence of the complete N-terminal strand in CanA (Table 3).

### Nanobiotechnological applications

Because of the ability of CanA to form heat-resistant, stable tubes of relatively large inner diameter (20 nm) that form spontaneously in the presence of divalent ions, CanA nanotubes have been recognized as promising biotechnological entities. In their patent Barton et al.^27^ proposed a variety of applications using either pure CanA or CanA chimeras with other proteins, nucleic acids, and polysaccharides. Typical example applications they proposed were (1) targeted drug delivery where the drug is encapsulated by CanA, (2) formation of macroscopic fibers, (3) use for stereoselective purification in HPLC, (4) production of biochips, (5) production of hydrogels, (6) fusion proteins with active enzymes. At the time of the patent, the 3D-structures of CanA monomers and polymers were not known. Therefore, it was not clear where catalytically active proteins or recognition sequences could be fused into the CanA-protein without deteriorating its cannulae formation. From the now elucidated 3D structures it is likely that the subdomain containing β-strands β_9_ and β_10_ could be replaced by sequences of the fusion partners (see Figs. 4, 17, 18). The fusion partners would be located outside the cannulae. Modification of the inner surface of the cannulae (Figs. 4, 17, 18) appears less obvious since a modification of the large β-sheet may destabilize the whole structure. A possible candidate for modifications would be the loop connecting β-strand β_7_ and β_8_. However, these assumptions have to be tested experimentally in a design study. In addition, the N-terminally truncated K_1_-CanA could be used to limit the growth of the cannulae in biochip applications.

## Conclusion

CanA forms a non-canonical jellyroll class I fold that has not been described in this form in other proteins of the database. In its monomeric form, it has probably 5 specific Ca^2+^ binding sites with *K*_D_ -values in the range 0.8 and 28 mM. At a salinity of 3.5%, reference sea water contains 10.5 mM Ca^2+^ (IASPO), meaning that all sites with *K*_D_-values smaller 10.5 mM should at least be half saturated. NMR data show that the binding of divalent ions induces significant structural changes that may initiate the polymerization. Most probably, they also release the N-terminal stretch of the sequence (1 to 22) from its interactions with the main body of the protein, allowing its interaction with other CanA subunits, which are required for stable polymerization. The structural changes in these regions are also confirmed by a comparison of the NMR structure of a protomer with its structure in the polymer obtained by cryo-EM. In the polymer, the Ca^2+^-ions are bound to similar sites as the monomers but also amino acids from adjacent monomers are involved in the binding. These contacts may strongly increase the affinity for Ca^2+^-ions in the polymer. Neutralization of the negative charges in the two binding areas BA1 and BA2 by positively charged divalent ions is also mandatory for the interaction of the three essentially uncharged N-terminal segments (except for their positively charged N-terminal NH_3_^+^-group) from neighboring protomers with surface residues of these regions.

## Methods

### Protein expression and purification

CanA from *Pyrodictium abyssi* and K_1_-CanA, its N-terminally truncated form, were expressed in *E. coli* and purified as described by Kreitner et al.^10^. Uniformly ^15^N- and ^13^C, ^15^N- labeled proteins were obtained by growing the bacteria in isotopically labeled New Minimal Medium (NMM)^28^, pH 7.4, containing 1 g/L ^15^NH_4_Cl or 1 g/L ^15^NH_4_Cl and 2 g/L ^13^C-glucose, respectively. For NMR spectroscopy, freeze-dried CanA or K_1_-CanA was dissolved in 20 mM sodium phosphate buffer (Na_2_HPO_4_/NaH_2_PO_4_, pH 6.6) containing 0.1 mM EDTA, 0.4 mM NaN_3_, 0.4 mM DSS and either 90% H_2_O/10% D_2_O or 100% D_2_O. The final concentration of CanA or K_1_-CanA was typically 0.1–1.0 mM dependent on the experiment performed.

### Transmission electron microscopy (TEM)

The samples used for the detection of cannulae contained 2 mM CanA in buffer A (50 mM Tris/HCl, pH 6.5, 80 mM NaCl, 0.4 mM NaN_3_) supplemented by 10 mM MgCl_2_, and 10 mM CaCl_2_. The typical polymerization time was 12 h at 303 K. Before blotting the sample onto a carbon-coated Cu grid, the protein was diluted by the addition of buffer A to an end concentration of 0.02 mM. Negative staining was done by application of 2% uranyl acetate (pH 4.7) to the sample. Transmission electron micrographs were digitally recorded using a 1k x 1k slow-scan charge-coupled device camera (TVIPS GmbH, Gauting, Germany), using a Philips CM12 transmission electron microscope, operated at 120 keV.

### FT-IR spectroscopy

The FT-IR spectra of non-polymerized and polymerized CanA were recorded with a Varian 670 FT-IR spectrometer. Polymerization was induced by the addition of 10 mM CaCl_2_ and 10 mM MgCl_2_. A highly concentrated protein film was used (1 mg in 5 µL H_2_O).

### CD spectroscopy

CD spectra of CanA and K_1_-CanA were recorded with a Jasco J-815 CD spectrometer at 298 K and analyzed with the program CDSSTR^13^. The typical concentration of the proteins was 1 mg/mL dissolved in H_2_O, pH 7.0.

### NMR spectroscopy

The NMR data were recorded on Bruker Avance-600 and Avance-800 spectrometers, equipped with a cryoprobe, at a temperature of 313 K and 323 K. For sequential backbone and side chain assignments heteronuclear experiments HNCA, CBCA(CO)NH, CBCANH, HNCO, [^1^H-^15^N]-HSQC, HCCH-TOCSY, and [^1^H-^13^C]-HSQC were performed at 313 K for full-length CanA and 323 K for K_1_-CanA. For the structure calculation of K_1_-CanA, we recorded homonuclear ^1^H-NOESY and heteronuclear ^1^H-^15^N-NOESY-HSQC spectra at 323 K. The interaction with divalent ions was studied by adding these ions in suitable concentrations and by observing the chemical shift changes by [^1^H-^15^N]-HSQC and/or [^1^H, ^15^N]-SOFAST-HMQC. The proton shifts were referenced to the ^1^H resonance frequency of the methyl group of DSS, and the ^13^C and ^15^N resonances were indirectly calibrated following the IUPAC recommendations^29^. The data were processed using the program Topspin (Bruker, Karlsruhe) and evaluated using the program AUREMOL^30^ (https://auremol.de).

### Sequential assignments

We have already published the complete homo- and heteronuclear assignment of uniformly ^15^N and ^13^C enriched K_1_-CanA in 20 mM sodium phosphate buffer (Na_2_HPO_4_/NaH_2_PO_4_, pH 6.6), containing 0.1 mM EDTA, 0.4 mM NaN_3_, 0.4 mM DSS in 90% H_2_O/10% D_2_O at 323 K^10^. Except for the N-terminal residues, the assignments of K_1_-CanA can be directly transferred to full-length CanA (see text).

### Structure calculation

Experimental distance restraints were obtained from an analysis of 2D- and 3D-NOESY spectra. Correlation peaks were picked, assigned, and integrated using the program AUREMOL^30^. Interatomic distances were obtained from the cross-peak volumes normalized to a set of unambiguous peaks with covalently fixed interatomic distances. Dihedral angles were obtained from the ^15^N^H^, ^13^C^α^, ^13^C^β^, and ^13^C chemical shifts using the program TALOS-N^14^. Hydrogen bonds were identified according to their dihedral angle and NOE patterns^31^. All peptide bonds were assumed to be planar and in *trans*-configuration^10^.

The structural calculations were performed using a simulated annealing protocol and the CNS 1.1 program^32^. A total number of 1000 structures were calculated, and the 10 lowest energy structures were deposited in the protein data bank with the accession number 9HDI.pdb.

Diffusion measurements in water were performed with a stimulated echo sequence^33,34^ using bipolar diffusion gradient pulses^35^ for diffusion and a 3-9-19 pulse for water suppression (Bruker pulse program stebpgp1s191d).

### Fitting of ion binding and polymerization

The binding of divalent ions to K_1_-CanA or CanA may involve different binding sites *I* with different numbers *N*_i_ of ligands and probably also involves cooperative effects coupled to local structural rearrangements. The chemical shift change Δ*δ* = *δ* − *δ*_0_ of a given atom induced by the independent binding of *N* ligands can be described by

1 $$\Delta \delta = { }\frac{{\Delta \delta_{N} }}{{2NM_{T} }}\left( {K_{D} + c_{T} + NM_{T} - { }\sqrt {\left( {K_{D} + c_{T} + NM_{T} } \right)^{2} - 4NM_{T} c_{T} } } \right)$$

with *δ*_0_, *K*_D_, c_T_, and *M*_T_ the chemical shift in the absence of ligands, the microscopic dissociation constant, the total concentration of the ligand, and the total concentration of the protein, respectively. Δ*δ*_*N*_ is the chemical shift change when all ligand binding sites are occupied. Without the knowledge of the concentrations of free ligands, the system is experimentally underdetermined. We modeled the binding by Eq. 1 for each atom influenced significantly by ion binding separately (see Discussion).

### Evaluation of the NMR diffusion measurements

The apparent hydrodynamic radius *R*_*h*_(CanA) of CanA was calculated with the hydrodynamic radius *R*_*h*_(DSS) from DSS contained in the CanA sample from the ratio of the corresponding diffusion constants *D*(CanA) and *D*(DSS) with

2 $${R}_{h}\left(CanA\right)={R}_{h}\left(DSS\right)\frac{D(DSS)}{D(CanA)}$$

The hydrodynamic radius *R*_*h*_(DSS) of DSS has been determined in diluted aqueous solutions of DSS using the known diffusion constants of D_2_O. It is 0.346 ± 0.004 nm at 298 K^36^. The relative diffusion constants were obtained by fitting the intensity *I* as a function of the gradient strength using the relation

3 $$I={I}_{0} {e}^{-aD{G}^{2}}$$

with *I*_0_ the intensity at a gradient strength *G* of 0, *D* the diffusion constant, and *a* a constant in a given measurement.

### Representation of structures, estimation of surface accessibility, and interacting residues

The representation of 3D structures and protein surfaces, the secondary structure analysis as well as the estimation of the water-accessible surface were performed with MolMol^15^. The water-accessible surface was determined using a rolling sphere with a diameter of 0.14 nm. Residues were assumed to be surface residues when > 10% of their surfaces were water accessible. Interacting residues were identified as described by Schumann et al.^17^ based on the calculation of Δδ^comb^ as implemented in the program AUREMOL^30^.

## Supplementary Information

Below is the link to the electronic supplementary material.

Supplementary Material 1

## Data Availability

The NMR structures of K1-CanA are deposited in the Protein Data Bank (PDB) as well as the correspondent NMR data under the accession number 9HDI.pdb. Additional data supporting the findings of this study are available from the corresponding authors upon request.

## Abbreviations

NMR: Nuclear magnetic resonance
CD: Circular dichroism
TEM: Transmission electron microscopy
FT-IR: Fourier-transform infrared spectroscopy
DSS: 2,2-dimethyl-2-silapentane-5-sulfonate
DTE: 1,4-dithioerythritol
GdmCl: Guanidinium hydrochloride
