# Supplementary material for: Biophysical characterization and solution structure of the cannulae-forming protein CanA from the hyperthermophilic archaeon Pyrodictium abyssi
Source: Sci Rep. 2025 Aug 5;15:28563. doi: 10.1038/s41598-025-13242-6 (PMC12326000; doi:10.1038/s41598-025-13242-6)
Supplement: Supplementary file 1 — Supplementary Material 1 [file 41598_2025_13242_MOESM1_ESM.docx]

**Supplementary Information**

**Biophysical characterization and solution structure of the cannulae forming protein CanA from the hyperthermophilic archaeon *Pyrodictium abyssi***

Claudia E. Munte^1^, Raphael Kreitner^1^, Reinhard Rachel^2^, Karl O. Stetter^3^, Werner Kremer^1^, Hans Robert Kalbitzer^1^*

^1^Institute of Biophysics and Physical Biochemistry, Biophysics I and Centre of Magnetic Resonance in Chemistry and Biomedicine (CMRCB), University of Regensburg, Universitätsstr. 31, D-93053 Regensburg, Germany.

^2^Centre for Electron Microscopy, University of Regensburg, Universitätsstr. 31, D-93053 Regensburg, Germany.

^3^Lehrstuhl für Mikrobiologie und Archaeen-Zentrum, University of Regensburg, Universitätsstr. 31, D-93053 Regensburg, Germany.

**Tables**

**Table S1: NMR restraints used for the structure calculation of K_1_-CanA at 323 K.**

| **Constraints** | **Number** |
| --- | --- |
| NOEs | 2474 |
| intraresidual (*i*, *i*) | 861 |
| sequential (*i*, *i*+1) | 637 |
| backbone-backbone | 293 |
| backbone-sidechain | 326 |
| sidechain-sidechain | 18 |
| intermediate range (*i, j; j < i+ 4* ) | 239 |
| backbone-backbone | 60 |
| backbone-sidechain | 112 |
| sidechain-sidechain | 67 |
| long range (*i*,*j*; *j* > *i+4*) | 737 |
| backbone-backbone | 208 |
| backbone-sidechain | 327 |
| sidechain-sidechain | 202 |
| φ,ψ-angle constraints | 318 |
| hydrogen bonds | 54 |
|  |  |
| total number of restraints | 2846 |
| Restraints/residue | 16.5 |

**Table S2: Structural statistics of the 10 lowest energy structures (from 1000) of K_1_-CanA at pH 6.6 and 323 K**.

| **Energy** | **[kJ mol^-1^]** |
| --- | --- |
| *E*_total_ | 5140.6 ± 258.8 |
| *E*_NOE_ | 2269.2 ± 190.6 |
| *E*_dihed_ | 190.0 ± 29.7 |
| *E*_bond_ | 70.4 ± 15.1 |
| *E*_angle_ | 1200.4 ± 82.9 |
| *E*_vdW_ | 952.9 ± 66.6 |
| *E*_imp_ | 231.5 ± 23.9 |
|  |  |
| **NOE Violations** |  |
| > 0.05 nm | 6.95 ± 1.65 |
|  |  |
| **RMSDs ^a^** | **[nm]** |
| whole K_1_-CanA | 0.094 (0.151) |
| core regions | 0.025 (0.087) |

^a^ All backbone atoms, values in brackets all non-hydrogen atoms.
